# The Hardness of Additively Manufactured Alloys

**DOI:** 10.3390/ma11112070

**Published:** 2018-10-23

**Authors:** J.S. Zuback, T. DebRoy

**Affiliations:** Department of Materials Science and Engineering, The Pennsylvania State University, University Park, PA 16802, USA; jsz125@psu.edu

**Keywords:** additive manufacturing, microstructure, hardness, mechanical properties, aluminum alloys, Steels, nickel alloys, titanium alloys

## Abstract

The rapidly evolving field of additive manufacturing requires a periodic assessment of the progress made in understanding the properties of metallic components. Although extensive research has been undertaken by many investigators, the data on properties such as hardness from individual publications are often fragmented. When these published data are critically reviewed, several important insights that cannot be obtained from individual papers become apparent. We examine the role of cooling rate, microstructure, alloy composition and post process heat treatment on the hardness of additively manufactured aluminum, nickel, titanium and iron base components. Hardness data for steels and aluminum alloys processed by additive manufacturing and welding are compared to understand the relative roles of manufacturing processes. Furthermore, the findings are useful to determine if a target hardness is easily attainable either by adjusting AM process variables or through appropriate alloy selection.

## 1. Introduction

Additive manufacturing (AM) involves the layer-by-layer deposition of alloys from powder or wire feedstock by rapid heating, melting, solidification and cooling. Evolution of microstructure and properties of the components during AM is affected by repeated thermal cycles, large temperature gradients and relatively high cooling rates during solidification and solid-state phase transformations upon cooling. Serviceability of additively manufactured components depend on their chemical composition, microstructure, properties and defects. Hardness is one of the most commonly tested mechanical properties because measurements are quick, relatively inexpensive and provide insight to other properties such as yield strength [1] and wear resistance [2]. Literature data on some mechanical properties such as the yield strength of additively manufactured components often show significant scatter that can mostly be attributed to the presence of internal defects. In contrast, micro-hardness data are largely unaffected by internal defects. As a result, hardness measurements indicate the true effect of microstructural features such as the presence of various phases, precipitate particles, average grain size and alloy composition. For a specific alloy, these microstructural features depend on the AM processing conditions.

Much of the reported mechanical property data in the literature are for as fabricated condition without any post processing heat treatment. Understanding of the hardness data for these cases allow examination of the role of AM process variables prior to the property improvements during post processing. During AM, cooling rates between the liquids and solidus temperatures and in lower temperature ranges where important solid-state phase transformations take place are rapid. As a result, there is often insufficient time for the fabricated components to attain equilibrium microstructures. In other words, the rapid thermal cycles often limit the extent of some of the phase transformations. In those situations, the data from multiple sources available in the literature allow us to examine the impact of the AM variables on the hardness variation and assess the extent to which AM processes allow tailoring of hardness of components immediately after their fabrication. Comparing these reported variations from independent studies with the known variations of hardness owing to changes in alloy compositions through materials selection enables a practical way to select both the alloy and AM process variations to achieve a target hardness.

Many of the engineering alloys used in AM require post processing to achieve optimal properties. Much attention has been given to adjusting process variables [3,4], optimizing scanning strategies [5,6], numerically calculating important metallurgical variables [7,8,9,10,11,12] and post-fabrication techniques like heat treatments or hot isostatic pressing [13,14] for achieving target properties. For example, it has been observed experimentally that the AM of age-hardenable alloys, which rely on the presence of small precipitates for strength and hardness, often require post-processing heat treatments that allow time for precipitation to achieve properties similar to those found in conventional processes [15]. For these alloys, heat treatment produces significant changes in microstructure and properties for a given composition of an alloy.

Although the serviceability of the components produced by AM depend on their microstructure and mechanical properties, the available data are fragmented in many individual publications. After only about a quarter of a century of research, there have been many influential research articles and critical reviews [16,17,18,19,20,21,22,23,24,25,26,27,28] addressing the scientific and technological advancements in the AM of metals. Moreover, several reviews have focused on mechanical properties [29,30,31,32], microstructures [30,33,34] and specific AM processes [35,36,37,38,39,40] and alloy systems [29,31,38,41,42,43,44]. A periodic assessment of the links between processing, microstructure and properties is needed to advance our understanding as AM is still a relatively new and rapidly developing fabrication method. This review provides a compilation of the available hardness data for AM components for widely used alloys in order to seek reusable insights and make conclusions that cannot be made from individual papers. These data allow us to examine the hardness variations from various AM processes characterized by different cooling rates and processing conditions.

Here we examine the role of metallurgical variables like cooling rate, microstructure, alloy composition and post-processing heat treatments on the hardness of multiple alloy components fabricated by AM. The data reviewed allow us to examine the role of AM process variants on the microstructure and component hardness prior to post processing heat treatment. In many papers, hardness values are reported without any microstructural characterization precluding any direct correlation between microstructure and hardness. In those cases, selection of an effective compositional variable allows examination of the role of important alloying elements on hardness. While such correlations cannot take advantage of the decades of research correlating microstructure with properties, they reveal several immensely useful insights. Furthermore, the findings discussed are useful to determine if a target hardness is attainable either by adjusting AM process variables or selection of an appropriate alloy composition. Finally, they serve as a basis for alloy selection for attaining a target hardness of a component fabricated by AM.

## 2. Influence of Process Variables

AM processes generally fall into two main categories depending on the delivery method of the feedstock material. Powder bed fusion (PBF) consists of spreading a fine layer of powder material across a substrate with a recoating blade or roller. The powder feedstock remains stationary while a heat source is scanned along predefined paths to produce the desired part in a layer-by-layer manner. In directed energy deposition (DED) AM, either powder or wire feedstock is delivered coaxially with the heat source into a molten pool. In contrast to PBF, the feedstock delivery system moves with the heat source in DED processes. Types of heat sources commonly used include lasers (L), electron beams (EB), plasma arcs (PA) and gas metal arcs (GMA). In this review, a distinction between individual studies will be made by defining the specific AM process and type of heat source. For example, a laser based DED process is designated as DED-L. There is a wide range of process parameter combinations used in different AM processes that determine the structure and properties of fabricated components. In this section, the influence of these variables on the hardness of AM alloys will be discussed.

### 2.1. Energy Input

The thermal histories during AM vary both spatially and temporally. Temperature measurements are limited to specific locations within the substrate when thermocouples are used and the surface of the molten pool when infrared imaging is used. Therefore, it is often difficult to represent the cooling rates and thermal histories of the entire AM process with a single value. An approximate alternative is to compare studies based on the amount of energy is delivered to the deposit in the form of a linear heat input [17]

(1) $$E=\frac{P}{v}$$

where *P* is the power of the heat source in Watts and *v* is the scanning speed in mm/s. While more complex expressions for energy input exist in the literature, they often contain more process variables for the calculation, the details of which are not always reported.

**Figure 1:**
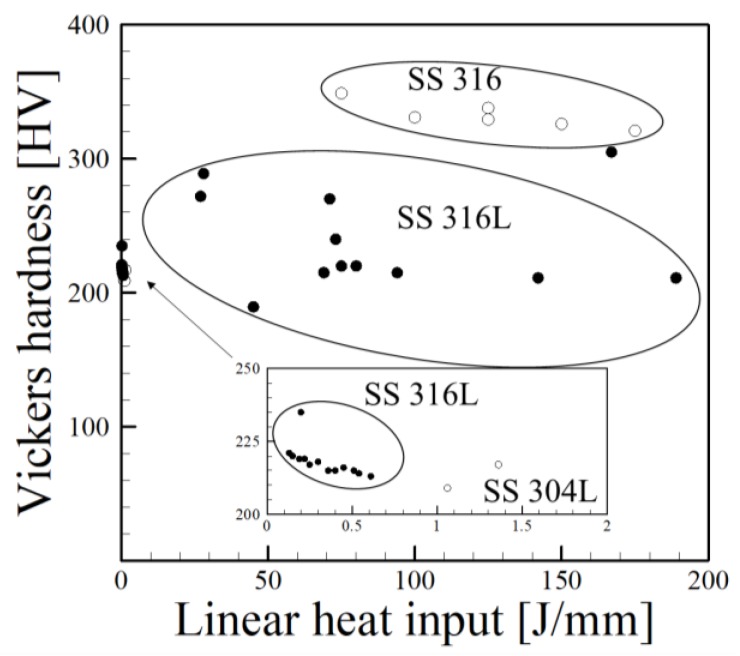
Hardness measurements for austenitic stainless steels deposited by AM as a function of linear heat input [45,46,47,48,49,50,51,52,53].

Austenitic stainless steels, such as SS 304L and 316L and titanium alloy Ti-6Al-4V have received much attention in the AM literature. Figure 1 shows the reported Vickers hardness [45,46,47,48,49,50,51,52,53] for austenitic stainless steels SS 316, SS 316L and SS 304L as a function of linear heat input. Generally, a higher heat input results in large molten pools, higher peak temperatures and slower cooling rates. It is expected that hardness will decrease with increases in linear heat input due to more heat accumulation, larger grain sizes and microstructural coarsening. A slight downward trend is observed in Figure 1 when all data is collected and plotted together. It should be noted that the scatter in the data can be caused by differences in equipment and techniques from independent researchers. However, it is interesting to note the differences with slight changes in chemical composition between SS316 and SS316L. Although each stainless steel is microstructurally similar, noticeable changes in micro-hardness are observed due to slight variations in carbon concentration.

**Figure 2:**
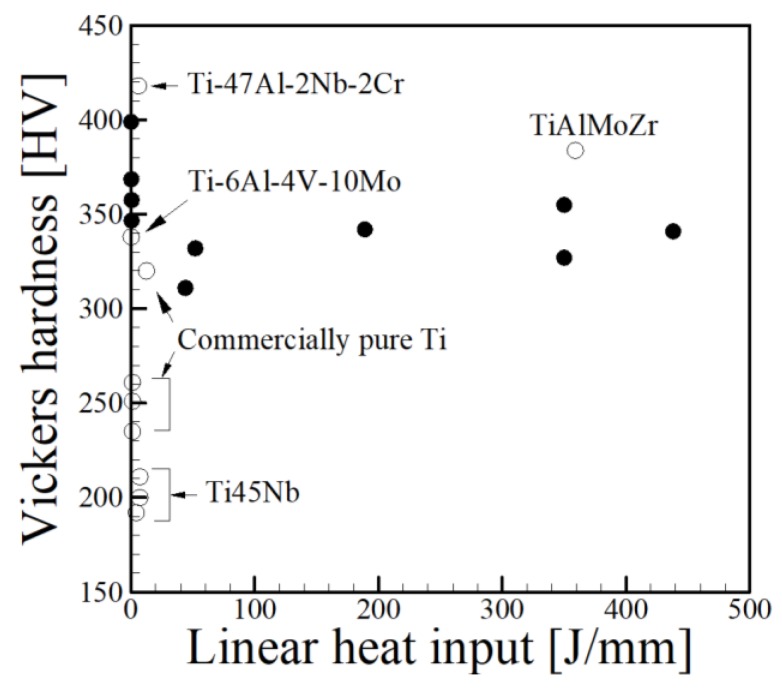
Vickers hardness as a function of linear heat input for titanium alloys where solid black dots correspond to Ti-6Al-4V and open points are marked otherwise [54,55,56,57,58,59,60,61,62,63,64,65].

In a similar manner, average hardness values [54,55,56,57,58,59,60,61,62,63,64,65] are plotted as a function of linear heat input for Ti-base alloys in Figure 2. Small values of linear heat input typically are found in powder bed processes where low powers and high scanning speeds are used. In contrast, DED and wire-based AM processes tend to use higher powers and lower scanning speeds, contributing to higher linear heat inputs. Observed microstructures of Ti-6Al-4V builds depend on the process used and process variables resulting in different cooling rates. Although there have been individual studies that show decreases in hardness with increases in linear heat input for a single process, no observable trend can be seen when all data is combined. However, the highest values are recorded for low linear heat inputs, which correspond to powder bed processes.

The use of linear heat input as a process variable has also been used in many studies to determine part density or residual porosity. It is generally accepted that an increase in the density of an AM component will effectively increase hardness measurements. Indeed, multiple investigations [52,63,66,67] have reported positive correlations between part density and hardness. However, it is important to note that most of these studies use a matrix of experiments where multiple process parameters are varied to optimize part density by reducing porosity. Changing process parameters that affect important metallurgical variables like cooling rates and molten pool geometry will often lead to changes in micro-hardness regardless of residual porosity. In fact, studies that contradict the correlation between part density and micro-hardness have also been reported [54,68]. For example, Thijs et al. [68] showed no apparent trend between part density and micro-hardness. After PBF-L of Ti-6Al-4V for a constant linear heat input of 0.21 J/mm, a sample with 99.6% density exhibited a Vickers hardness of 409 HV while a value of 426 HV was measured for a sample with approximately 96% density. These changes in micro-hardness were caused by a difference in hatch spacing that ultimately affects cooling rates and resulting microstructure.

**Figure 3:**
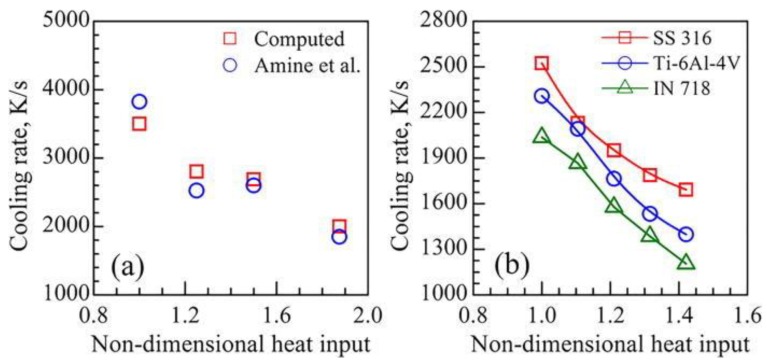
The relationship between computed cooling rates and a dimensionless heat input parameter for the DED-L of (**a**) SS316L validated from experimental data [70] and (**b**) common AM alloys under typical process conditions [69]. Reprinted from [69] with permission from AIP Publishing.

The heat input for a given set of process conditions can be linked to the cooling rate. Larger heat inputs generally result in slower cooling rates due to the large molten pool sizes and higher peak temperatures. Mukherjee et al. [69] used a 3D heat transfer and fluid flow model to show that the computed cooling rates have an inverse relationship with a dimensionless heat input, that is, low heat inputs yield high cooling rates and vice versa. The dimensionless parameter is similar to Equation (1) and was defined as Q* = (P/v)/(P_R_/v_R_) where *P* and *v* are the laser power and scanning speed, respectively. The terms *P_R_* and *v_R_* represent the reference power and scanning speed taken to be the those that give the lowest heat input for the analyzed data set, making Q* always greater than unity. The computed cooling rates were validated with experimental data from Amine et al. [70] for the multilayer DED-L of SS316L as shown in Figure 3a. The calculations were then further extended to show the relationship between heat input and cooling rates for other common AM alloys in Figure 3b under typical processing conditions. For all cases, similar downward trends of cooling rates are observed for higher non-dimensional heat inputs.

### 2.2. Cooling Rates

**Figure 4:**
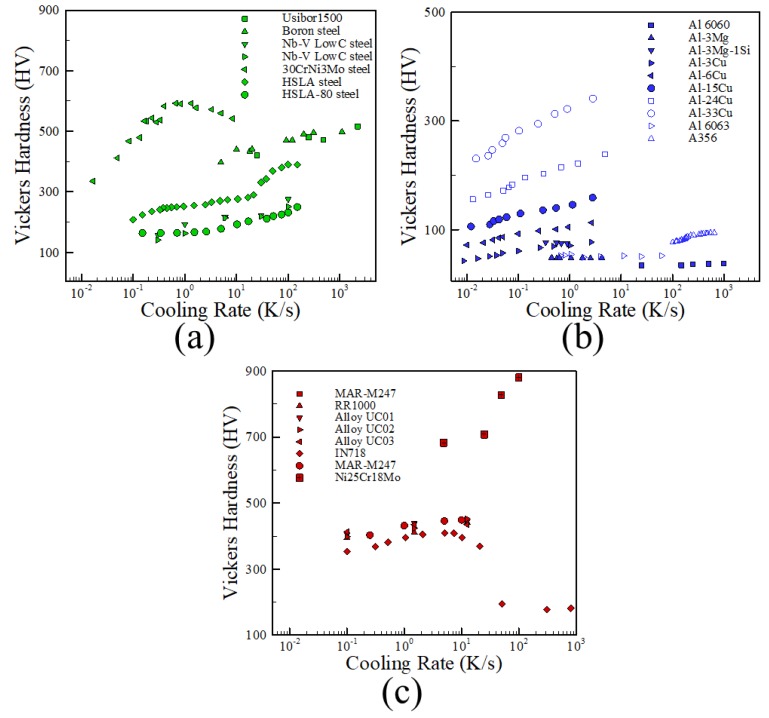
Hardness data as a function of reported cooling rates for (**a**) steels [71,72,73,74,75,76], (**b**) aluminum alloys [77,78,79,80,81] and (**c**) nickel alloys [82,83,84,85,86] in which no post-processing heat treatment was used.

In conventional metals processing, desired microstructures and properties are achieved through precise control of cooling rates and subsequent heat treatments. The controlled cooling rates of bulk materials are approximately spatially uniform and lead to repeatability in microstructure and properties for an alloy of a given chemical composition. For this reason, useful correlations can be developed that directly relate the micro-hardness of an alloy to cooling rate. Figure 4a–c shows such relationships between hardness and cooling rates for collected data on steels [71,72,73,74,75,76], aluminum alloys [77,78,79,80,81] and nickel alloys [82,83,84,85,86] in which plates or bars are cooled at controllable rates. The logarithmic scale on the horizontal axis shows that the cooling rates cover multiple orders of magnitudes. For each of the alloy classes, similar symbols indicate alloys of the same composition. Moving from left to right on the plot for a single composition indicates a change in hardness for an alloy due to an increasing cooling rate whereas moving vertically along the plot for any given cooling rate compares hardness changes due to a change in composition for alloys of the same class. Also, it can be seen that in most cases hardness values tend to plateau at high cooling rates for all alloy systems considered.

**Figure 5:**
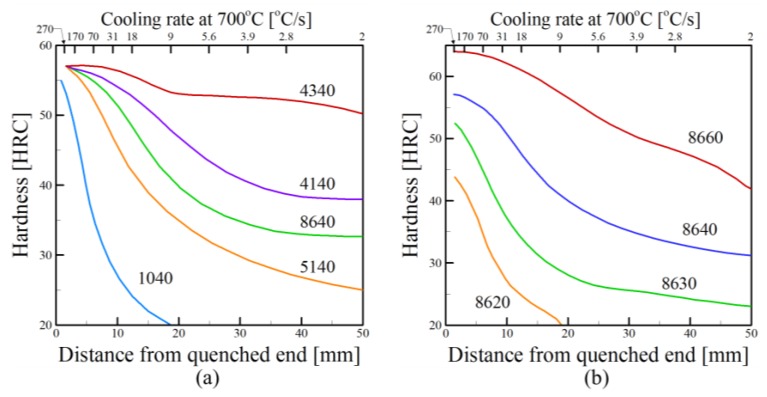
Hardness data for Jominy end quench experiments for (**a**) various steels with similar carbon concentrations and (**b**) 8600 series steels (0.55Ni, 0.50Cr, 0.20Mo) with varying carbon concentrations [87]. Reprinted from [87] with permission from Wiley.

From Figure 4a–c, it is observed that hardness differences resulting from changes in cooling rates are greatly outweighed by those when comparing alloys in the same class with different compositions. This trend is further supported by Figure 5 which shows hardness data for Jominy end quench samples for various grades of steel [87]. In Figure 5a, a comparison is made between different types of alloy steels with similar carbon concentrations. Similarly, Figure 5b compares 8600 series steels, having small amounts of Ni, Cr and Mo, with varying carbon contents. In both figures, changes in hardness due to both differences in chemical composition and cooling rates is substantial. Furthermore, increases in carbon concentration represented in Figure 5b can result in significant increases in hardness, regardless of cooling rate.

**Figure 6:**
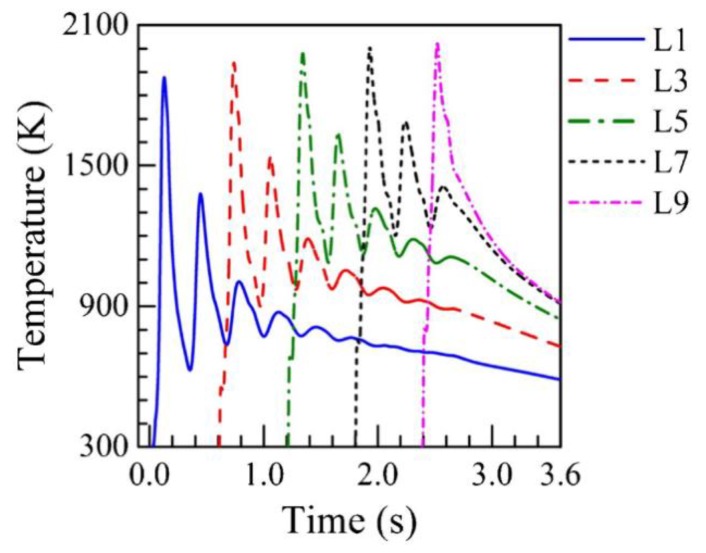
Computed thermal histories at the mid-length and mid-heights of selected layers during a single pass, nine layer simulation of DED-L of SS316 [8]. Reprinted from [8] with permission from Taylor & Francis.

The thermal histories in AM involve multiple cycles of rapid heating and cooling and can span multiple orders of magnitude depending on the process and temperature range at which the cooling rate refers to. Figure 6 shows numerically computed temperature cycles from a heat transfer and fluid flow model during DED-L of SS316L for a single track, nine layer deposit [8]. Monitoring locations were selected at the midpoint in the length and width directions for selected layers. Clearly, cooling rates vary drastically both as a function of time and location within a build. For this reason, cooling rates are difficult to quantify for multi-pass, multi-layered AM parts that experience repeated heating and cooling and can make microstructural analyses convoluted.

Both experimental and computational efforts have been undertaken for simple AM builds consisting of single passes and few layers to understand the relationship between cooling rates and micro-hardness for austenitic stainless steels. During the DED-L of SS316L [88], it was shown experimentally that the average cooling rates ranged from 22–764 °C/s for different processing conditions. The corresponding Vickers hardness values measured were approximately 150 HV for the slowest cooling rate and 368 HV for the highest cooling rate. Although it is unclear of the temperature range at which the cooling rate was averaged, the high hardness value was attributed to the formation of martensite. Manvatkar et al. [9] combined a numerical heat transfer and fluid flow model with experiments to correlate cooling rates to micro-hardness for a single pass, three layer DED-L deposition of SS316. The calculated cooling rates, defined as the average cooling rate through the solidification temperature range, varied from approximately 7000 °C/s in the first layer to 3000 °C/s. The resulting measured hardness values were approximately 230 and 210 HV for the first and third layers, respectively.

Another important process variable that is often overlooked when developing correlations is the geometry of the deposited part. The thermal history at an arbitrary location within a build will depend on the melting and solidification of material around that location. Also, the heat transfer conditions that govern the temperature history can change as different part geometries are used. Although no simple universal expressions exist to quantify the effects of geometry, a systematic study by Keist and Palmer [1,89] investigated the effects of geometry on mechanical properties of Ti-6Al-4V fabricated by PBF and DED using both laser and electron beam sources. When comparing measurements between thin and thick L-shaped walls, the Vickers hardness numbers of single pass walls were significantly lower than the hardness of the 3-pass walls.

**Figure 7:**
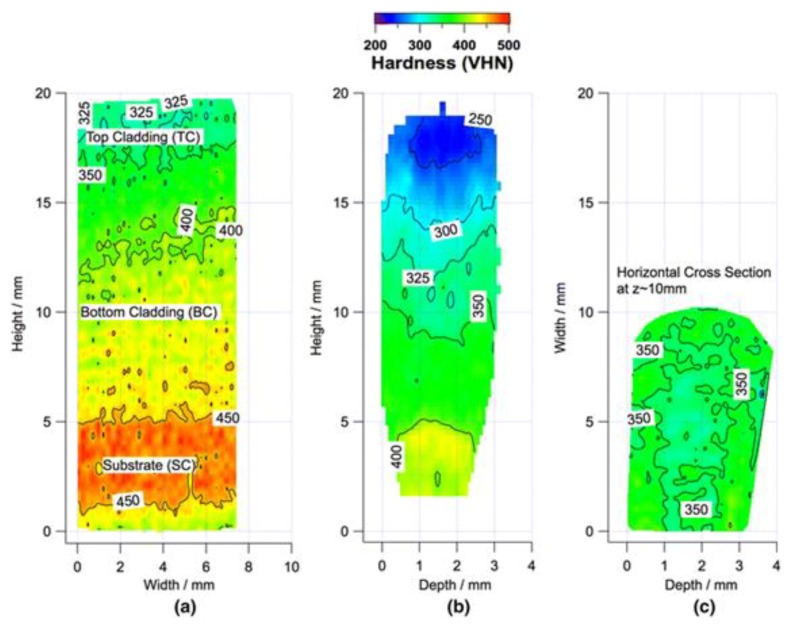
Hardness variations as a function of location within a DED-L single pass, multilayer build of IN718 [90] showing (**a**) a longitudinal cross section (X-Z plane); (**b**) a transverse cross section (Y-Z plane) and (**c**) a horizontal cross section (X-Y plane) where X is the travel direction, Y is the track width direction and Z is the build direction. Reprinted from [90] with permission from Springer Nature.

In geometries consisting of simple shapes, the hardness variations can easily be correlated with location in a build by taking multiple cross sections. In general terms, heat accumulates in a build with an increase in process time. As the build height increases further away from the substrate material, the heat transfer is directed through previously deposited layer which usually results in slower cooling rates. Extended times at elevated temperatures for alloys that are not precipitation hardenable causes coarsening of microstructural features, relaxation of residual stresses and dislocation motion and annihilation. As a result, hardness measurements tend to be lower at locations further away from the substrate (increasing build height) and higher in areas close to the substrate. Figure 7 shows cross sections with different orientations with respect to the build for a single pass wall of IN718 processed with DED-L. The most obvious changes in micro-hardness occur in the Z-direction as shown in Figure 7a,b, which correspond to the build direction. No significant differences were observed in the X- and Y-directions as observed in Figure 7b,c. Since IN718 is a precipitation-hardened alloy, appreciable changes in hardness at different locations in the build were attributed to aging during processing [90]. Regions near the baseplate experienced more time at high temperatures leading to enhanced precipitation.

## 3. Effects of Microstructure

As-deposited microstructures of alloy components fabricated by AM are direct products of the thermal histories experienced during heating, melting, solidification and cooling. Microstructure evolution depends on alloy composition and some alloys undergo important phase transformations that can impact the properties and performance of AM parts. Table 1 shows a collection of process conditions for steels, aluminum and nickel alloys that have been fabricated by AM. Correspondingly, Table 2 shows the phases and hardness values reported for the same alloys. In this section, the influence of microstructures on the hardness of AM alloys is discussed below for various types of alloys.

### 3.1. Iron Based Alloys

Due to the high costs of AM compared to conventional casting and forging processes, the use of plain carbon and low alloy steels have not found significant usage for AM applications outside of a few studies [126,127]. Many of the steels used in AM have been tools steels [128,129,130,131,132,133,134], studied for specialized repair applications in which high strength and wear resistance is crucial. Tools steels such as M2 and H13 obtain high strengths and hardness due to their propensity to form martensite even at relatively low cooling rates. Additionally, these alloys contain high amounts of carbon which promote the formation of carbides and increase strength and hardness. Typical microstructures of tool steels processed by AM include a martensitic matrix with some carbide precipitation and retained austenite [129,134]. After deposition of tool steels, heat treatment is desirable to increase ductility and toughness. A tempered martensitic microstructure with carbide precipitates results from extended times at high temperatures [131].

**Figure 8:**
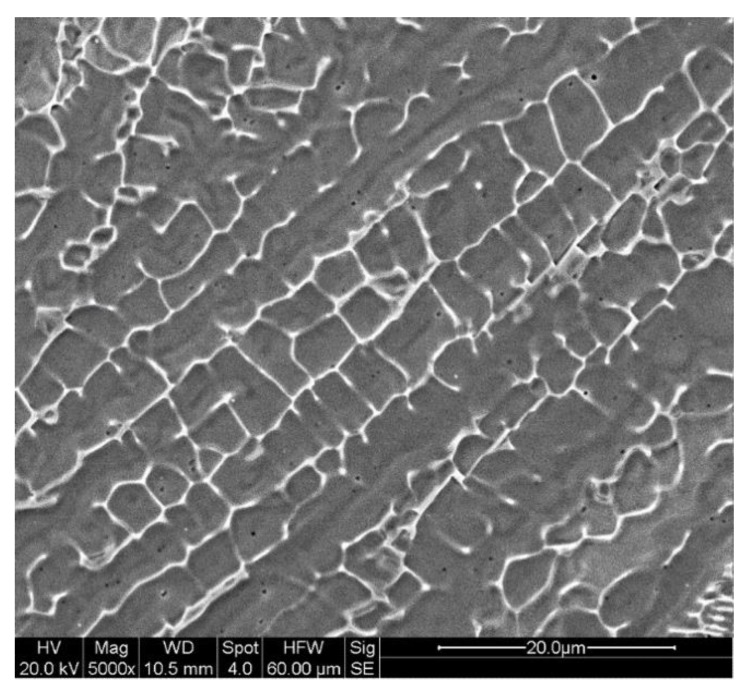
Columnar dendritic microstructure of SS316L deposited by DED-L [53]. Reprinted from [53] with permission from Elsevier.

Austenitic stainless steels, such as SS304L and SS316L exhibit predominantly austenitic microstructure consisting of cells and columnar dendrites, depending on the type of AM technique and the process parameters. Figure 8 shows an SEM micrograph of the typical columnar dendritic morphology of the austenitic grains encountered for SS316L fabricated using DED-L. Although austenitic stainless steels are dominated by an austenitic matrix, small amounts of delta ferrite can form as a result of thermal cycles and micro-segregation. High cooling rates during solidification favor austenite whereas lower cooling rates tend to yield increasing amounts of delta ferrite [135]. In PBF processes, a fine cellular solidification structure on the order of 1 μm is often observed. As austenitic stainless steels often do not precipitate secondary phases or undergo other solid-state transformations, the strength and hardness depend on the fineness of the solidification structure and chemical composition.

**Figure 9:**
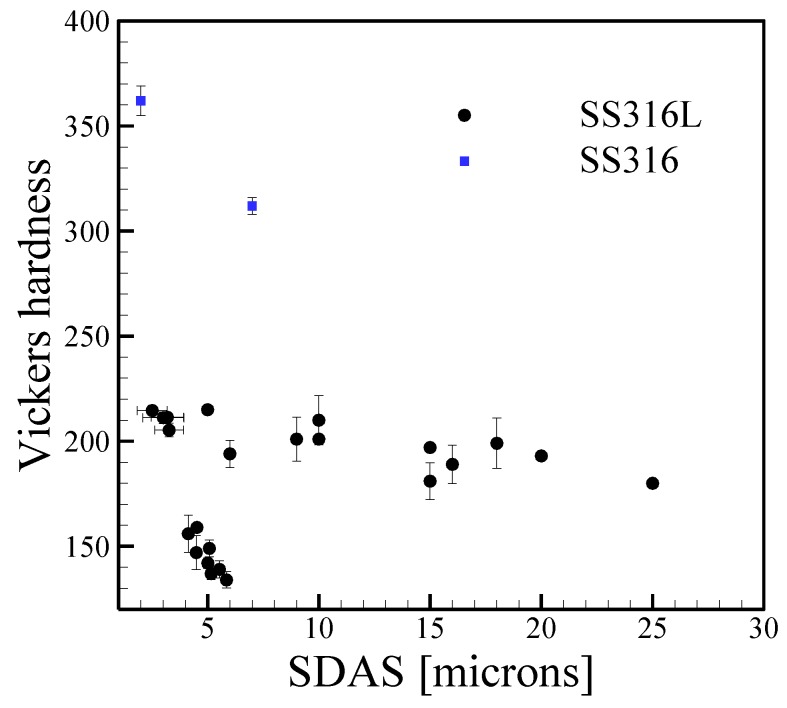
Vickers micro-hardness as a function of secondary arms spacing for stainless steels fabricated by AM from [45,53,70,136,137]. Error bars represent the standard deviation in measurements.

Figure 9 shows a collection of measured HV as a function of secondary dendrite arm spacing (SDAS) for the austenitic stainless steels SS316 and SS316L processed by DED-L. Both alloys typically exhibit large austenitic columnar grains with a dendritic substructure. Although these alloys are chemically similar, SS316L (≤0.03 wt % C) contains slightly less carbon than SS316 (≤0.08 wt % C) to help improve weldability and prevent sensitization. This small change in composition has pronounced effects on micro-hardness for roughly the same size SDAS with measurements for SS316, although limited, having a hardness of nearly 100 HV higher than those of SS316L. The same effect was observed in Figure 1. Therefore, it is important to perform detailed chemical composition analyses when studying the effects of microstructure on properties as small fluctuations in concentration of carbon can significantly affect hardness.

Precipitation hardened (PH) stainless steels have received considered attention in the AM community with the most commonly processed alloys being 17-4PH and 15-5PH. Nominal compositions for the PH grade stainless steels typically promote the formation of martensite during rapid cooling. Subsequent solutionizing and aging allows for Cu-rich nanoparticles to precipitate in a tempered martensite matrix which determine the final properties. However, microstructures ranging from austenitic/martensitic to primarily martensitic have been reported in the AM literature depending on the atomization condition of the powder feedstock and the type of shielding gas used. Multiple investigations [138,139] have shown that when Ar-atomized 17-4PH stainless steel was processed under an Ar atmosphere, a primarily martensitic structure was obtained. However, when nitrogen, an austenite-stabilizing element, was used as a processing gas to melt nitrogen atomized powders, a mixture of primarily austenite and martensite was observed. In the as-deposited conditions, substantial differences in micro-hardness in the two extreme conditions were measured, as the samples with more austenite exhibited a hardness slightly over 200 HV and the martensitic samples had a hardness slightly less than 400 HV [139].

The microstructure evolution during solutionizing and aging is highly dependent on the as-deposited microstructure. Standard solution heat treatments originally developed for wrought alloys may not be applicable due to the heterogeneous microstructures encountered in AM parts. For example, Cheruvathur et al. [140] found that the Vickers hardness of solutionized 17-4PH grade stainless steel was 312 ± 17 HV compared to a value of 258 ± 8 for the as-deposited condition. Although 17-4PH is classified as a martensitic stainless steel, a mixture of approximately 50% martensite and 50% retained austenite was found in the as-deposited condition with small amounts of NbC. After solutionizing and subsequent air cooling, less retained austenite was found in the microstructure, which was attributed to the increase in hardness.

### 3.2. Aluminum Alloys

**Figure 10:**
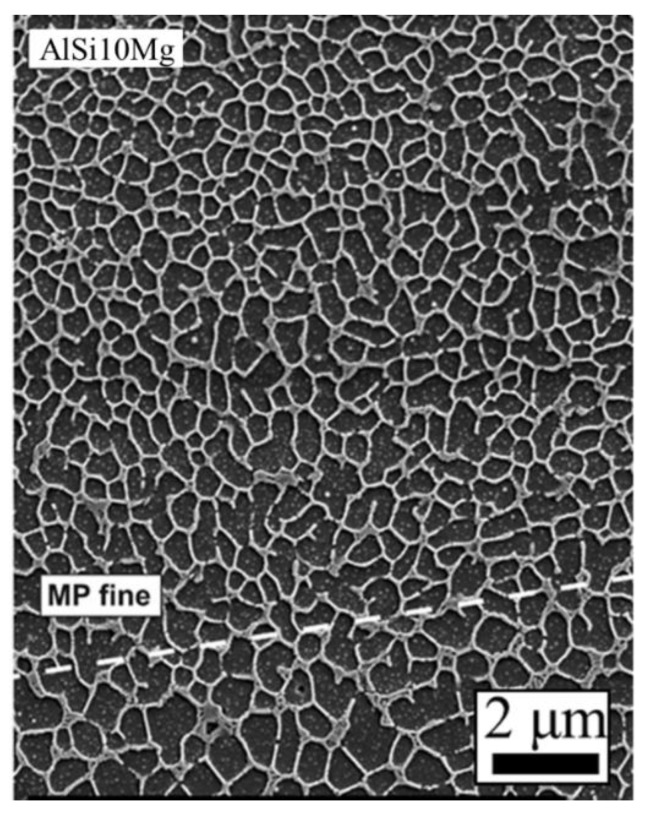
As-deposited microstructure of AlSi10Mg alloy fabricated by selective laser melting showing a fine cellular/dendritic structure with small amounts of eutectic [141]. Reprinted from [141] with permission from Elsevier.

**Figure 11:**
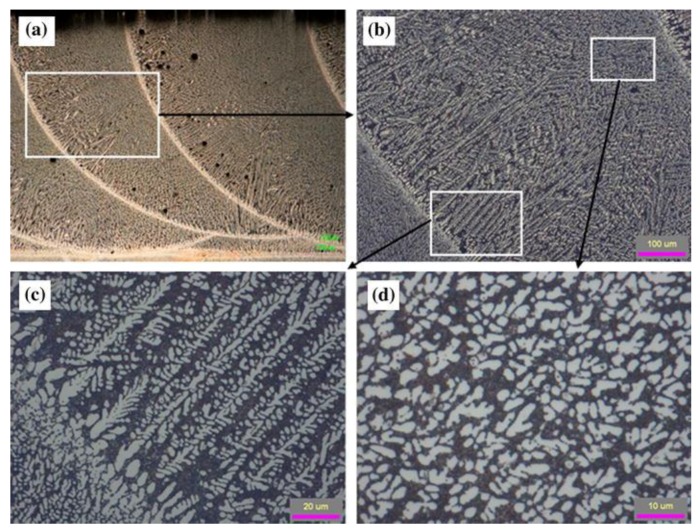
Microstructure of laser deposited Al 4047 showing dendritic and equiaxed structures at different locations within the same layer [142]. Reprinted from [142] with permission from Springer Nature.

The aluminum alloys most commonly used in AM processes contain large amounts of Si which promote eutectic solidification. The lower melting point of eutectic Al-Si alloys, such as Al-12Si and AlSi10Mg, are easier to process via laser-based AM processes compared to other aluminum alloys due to the low absorptivity of Al over a wide range of wavelengths. Aluminum alloys generally exhibit a cellular or dendritic microstructure consisting of a face centered cubic Al-matrix with fine Si-rich phases in the as-deposited condition when processed by AM. For example, Figure 10 shows cubic silicon phase in a fine cellular/dendritic structure within the face centered cubic aluminum matrix of AlSi10Mg after selective laser melting [141]. Eutectic formation was found to form at the triple points of the cellular/dendritic structure. Although cells and dendrites predominate in much of the literature, partial equiaxed microstructures [142] can be observed as shown in Figure 11 depending on the solidification parameters.

**Figure 12:**
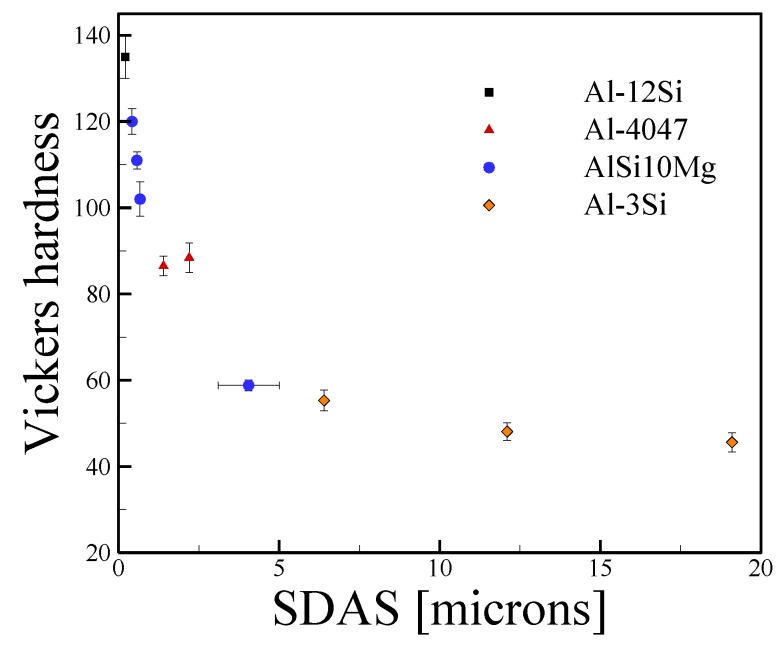
Vickers micro-hardness as a function of secondary arms spacing for aluminum alloys fabricated by AM from [112,142,144,145] and selected data for directional solidification of Al-3Si from Kaya et al. [143] for comparison. Error bars represent the standard deviation in measurements.

As precipitation hardenable aluminum alloys rely on aging heat treatments to achieve enhanced properties, the high strengths and hardness observed in as-fabricated alloys stem from the fine solidification structure. Although there is sparse data fragmented over multiple aluminum alloy systems, Figure 12 shows a collection of HV values plotted as a function of SDAS from AM. Selected data from directionally solidified aluminum alloys Al-3Cu, Al-1Ti and Al-3Si [143] is shown for comparison. A combination of high cooling rates and significant amounts of alloying elements, namely Si, lead to fine secondary dendrite arm spacing and high hardness in the alloys processed by AM. However, any attempts to link microstructure to properties should be limited to single compositions and Figure 12 merely shows a collection of data to demonstrate the fineness of AM microstructures in comparison to other processes. Any significant deviations in chemical composition that affect solidification requires separate analysis.

Although high strengths and hardness have been obtained after the AM of aluminum alloys, as-deposited components typically suffer from poor ductility. Investigations into the effects of post-process heat treatments on the mechanical properties of AlSi10Mg processed by PBF-L have resulted in interesting conclusions. Results from Aboulkhair et al. [146] showed that when applying standard T6 heat treatments (solutionizing + artificial aging) at various solutionization times, the Vickers hardness of heat treated samples (between 75–100 HV) was always less than the hardness in the as-deposited condition (~110 HV). Although Li et al. [147] reported similar findings, their results showed that the hardness after solutionizing was, in fact, greater than the hardness after artificial aging. After microstructural examination, it was found that Si particles formed during solutionization and subsequent artificial aging coarsened the particles to an extent similar to overaging in commonly processed aluminum alloys. However, Kempen et al. [148] achieved about a 12% increase from the as-deposited hardness (136 ± 9 to 152 ± 5 HV) when directly applying an artificial aging heat without a solutionizing step.

The use of HIP as a post-processing technique has found widespread use across many different alloys systems used in AM. In most cases, a dramatic effect can be observed when comparing the hardness of as-deposited and post-HIP conditions. During the PBF-L of AlSi10Mg [149], subsequent HIP treatment resulted in a hardness value (60 ± 5 HV) more than half of that of the as-deposited condition (125 ± 5 HV) due to significant microstructural coarsening and stress relief. Similarly, Tradowsky et al. [150] found that for machined AlSi10Mg samples fabricated by PBF-L, yield strength decreased by more than 60% after post-process HIP. However, the loss of strength was compensated by a substantial increase in percent elongation from approximately 5% in the as-deposited condition to about 21% after HIP.

### 3.3. Nickel Alloys

**Figure 13:**
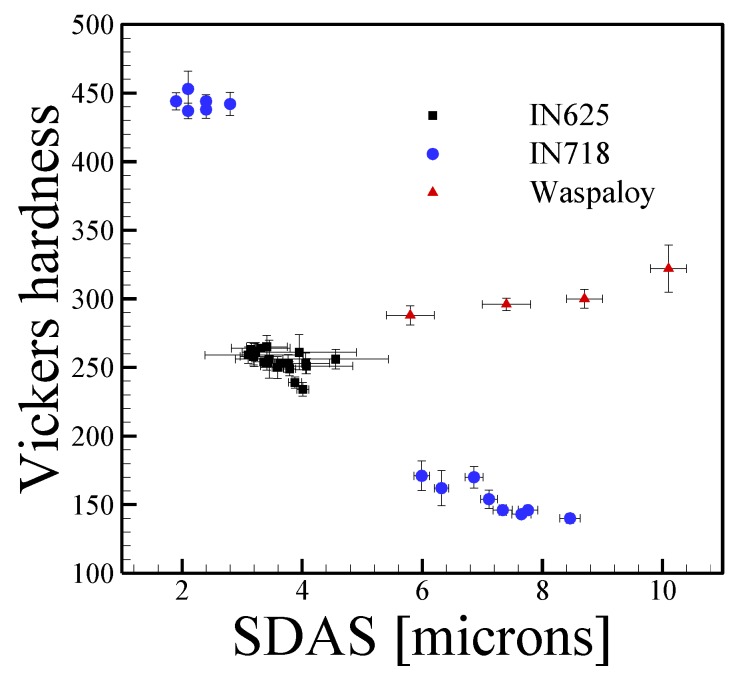
Vickers microhardness as a function of secondary arms spacing for nickel alloys fabricated by AM from [137,151,152,153,154,155]. Error bars represent the standard deviation in measurements.

Nickel alloys are some of the most complex alloys used in AM applications due to the large amounts of alloying elements that can result in various types of secondary phase precipitation which ultimately affect mechanical properties. The as-deposited microstructures of nickel alloys are highly dependent on thermal histories and chemical composition. Figure 13 shows microhardness as a function of SDAS for the DED-L of IN625, IN718 and Waspaloy. In the case of IN625, data points are confined to a rather tight grouping with an average micro-hardness and SDAS of approximately 250 HV and slightly less than 4 μm, respectively. Although IN625 is generally classified as a solid solution strengthened alloy, secondary phases often form in both welding and AM due to significant micro-segregation. However, these secondary phases (Laves, MC carbide) often form upon solidification and appreciable nucleation and growth in the solid state during AM processing is uncommon.

In the case of IN718, the highest HV values in Figure 13 correspond to a post-process heat treatment where the lower values were measured in the as-deposited state. It is clear from this comparison that the changes in hardness due to SDAS is negligible when compared to the effects of precipitation hardening. Interestingly in the case of the Waspaloy data, a positive correlation is observed between HV and SDAS. At first, this may seem counterintuitive, however the increases in SDAS were a result of deposits with different layer numbers with a higher number of layers corresponding to higher SDAS. As more heat was accumulated in the build and cooling rates decreased, microstructural coarsening occurred simultaneously with nucleation and growth of γ’ precipitates. Therefore, the observed hardness is contributed by both the coarsening as well as the precipitation of γ’ phase.

**Figure 14:**
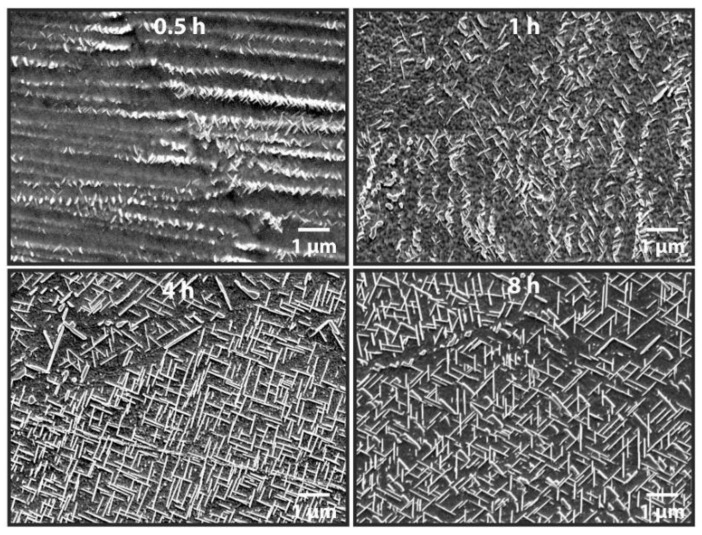
The precipitation and growth of δ phase at various times in PBF-L IN625 subjected to a standard stress relief heat treatment at 870 °C [156]. Reprinted from [156] with permission from Elsevier.

The as-deposited microstructures in AM alloys vary drastically from those of wrought counterparts for which standard heat treatments were developed. Consequently, large amounts of elemental segregation often observed in AM parts are expected to contribute to heat treatment responses that vary significantly from those of wrought parts. Zhang et al. [156] showed that after just five minutes at the manufacturer recommended stress relief temperature (870 °C), IN625 processed by PBF-L began to nucleate and grow deleterious δ-phase. Figure 14 shows the time evolution of the needle-shaped precipitates during stress-relieving. For comparison, an isothermal transformation diagram for wrought IN625 [157] does not predict the formation of δ-phase until approximately 10 h at 870 °C. The presence and morphology of intermetallic phases such as those shown in Figure 14 act as stress concentrators and can be detrimental to the ductility and toughness of materials. Instances such as these may prompt the need for developing standard heat treatments specially designed for AM materials to avoid undesirable microstructural evolution.

Vilaro et al. [115] reported hardness increases during 8 h stress relief heat treatments of PBF-L Nimonic 263 up to approximately 800 °C. Even though the residual stresses present in the as-deposited condition were greatly reduced at 600 °C, a slight increase in micro-hardness was observed. It was proposed that the high-density dislocation structure was unable to restore due to the presence of very small (<10 nm) γ’ particles that pinned dislocation motion at these temperatures which, along with the precipitation M23C6 carbides, increased hardness. It was not until a heat treatment above the γ’ solvus temperature (~960 °C) was used that the precipitates dissolved and the dislocation density was reduced.

**Figure 15:**
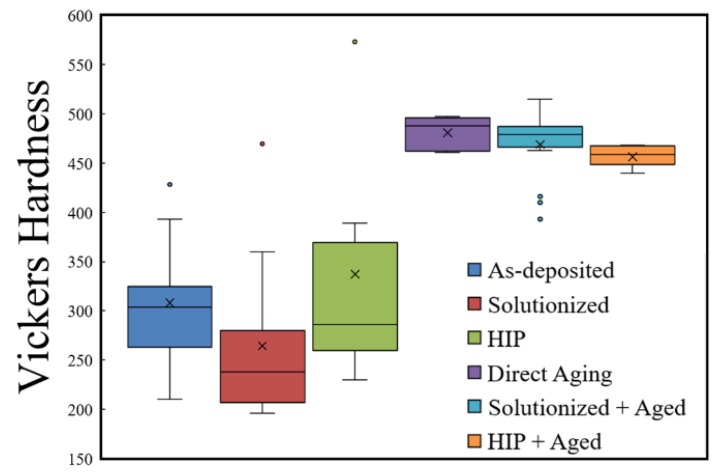
Box and whisker plot showing the variation in Vickers hardness of IN718 parts fabricated by DED-L [118,158,159,160,161], PBF-L [67,162,163,164,165,166,167,168] and PBF-EB [169,170] AM and subjected to various post process heat treatments.

Although IN718 is a nickel alloy, other elements like Al, Cr, Fe, Mo, Nb and Ti constitute nearly half of the alloy mass which aid in the nucleation and growth of γ’ and γ” precipitates. Although slight variations in time and temperatures have been used, post-processing of IN718 components fabricated by AM generally follow standard heat treatments including solutionization at 980 °C for 1 h followed double aging at 720 °C for 8 h and 620 °C for 8 h. The box-and-whisker plot in Figure 15 shows a collection of literature data for the hardness of IN718 parts at various stages of post-processing after AM fabrication. Individual points in Figure 15 represent outlying data. Clearly, significant advantages are gained in post-process heat treating to achieve properties that are otherwise unattainable in as-deposited parts.

**Figure 16:**
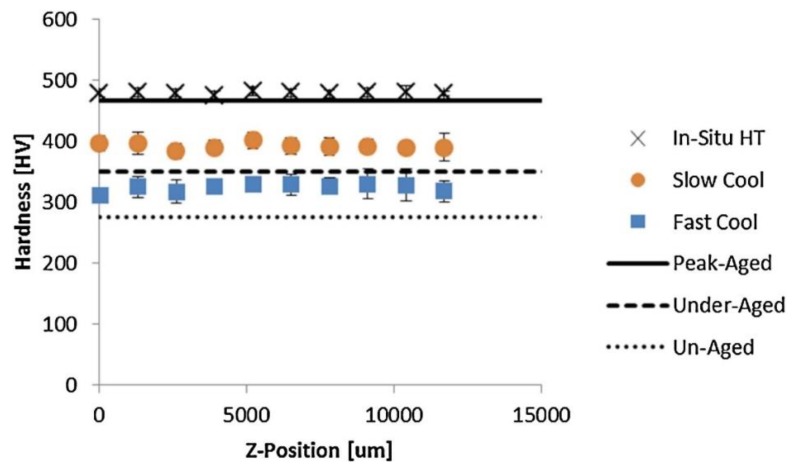
Measured Vickers hardness during PBF-EB of IN718 subjected to different cooling cycles and *in-situ* heat treatment [171]. Reprinted from [171] with permission from Elsevier.

An effort was undertaken by Sames et al. [171] to circumvent post-processing of IN718 while still achieving peak-aging properties. An *in-situ* heating method was used on a PBF-EB system where the build was consistently held at high temperatures to promote aging. A comparison of the Vickers hardness measured after the *in-situ* heat treatment and under fast and slow cooling is shown in Figure 16. Although the measurements showed that optimal hardness values were attainable, further tensile testing revealed significantly lower strength and elongation than control specimens due to the presence elongated cracks. In another study, Schwab et al. [172] used a similar *in-situ* heating method to enhance the properties of Ti-5553 during PBF-L through substrate heating. About a 60% increase in Vickers hardness was achieved and higher compressive strength was measured when comparing the heat-treated deposit to the samples with no substrate heating.

Khayat and Palmer [155] found for multiple IN625 feedstocks with varying iron contents fabricated by DED-L that HIP resulted in a Vickers hardness roughly 40 HV lower than the as-deposited material. Although the volume fraction of secondary phases increased during post-processing, which normally contribute to increased hardening, it is likely that the observation was outweighed by other factors like decreased dislocation density and elimination of the fine dendritic structure.

### 3.4. Titanium Alloys

**Figure 17:**
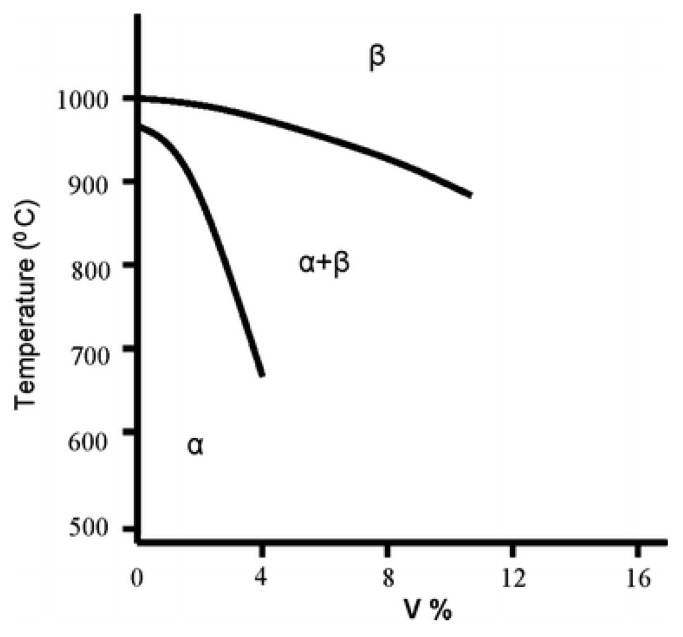
A portion of the Ti-Al-V phase diagram [173] for a constant aluminum concentration of 6 wt %. Reprinted from [173] with permission from Springer Nature.

**Figure 18:**
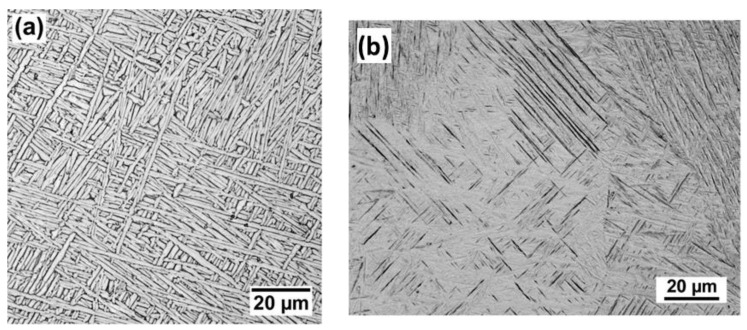
Representative micrographs of Ti-6Al-4V fabricated by (**a**) DED and (**b**) PBF after stress relieving [1]. Components fabricated by DED typically display coarse lamellar α-laths with small amounts of β while PBF components have much finer acicular martensite (α’). Reprinted from [1] with permission from Elsevier.

The combination of high strength and low density make titanium alloys an attractive alloy for AM in aerospace applications. The Ti-6Al-4V alloy is the most studied of the titanium alloys. The microstructure of Ti-6Al-4V consists of hexagonal close packed (α) and body centered cubic (β) phases as shown by the phase diagram in Figure 17. The addition of Al stabilizes α phase while V stabilizes β. Generally, as-deposited microstructures after powder bed fusion (PBF) AM exhibit a fine martensitic (α’) structure with acicular laths while the microstructures in directed energy deposition (DED) AM typically have a coarser structure consisting of lamellar α and small amounts of β [1]. Figure 18a,b shows representative micrographs of Ti-6Al-4V fabricated by DED and PBF AM.

**Figure 19:**
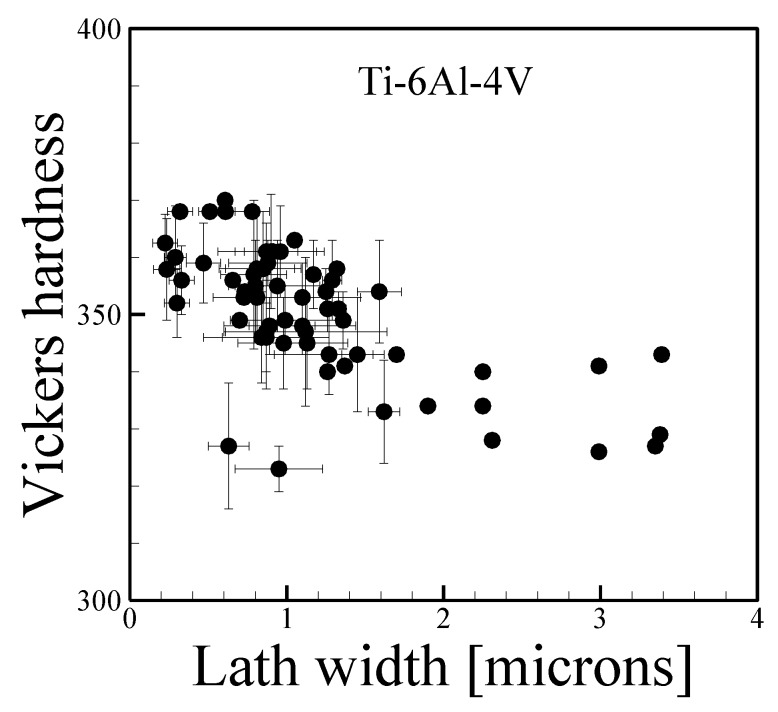
Vickers micro-hardness as a function of alpha lath width for Ti-6Al-4V fabricated by AM from [54,152,174,175,176,177,178,179]. Error bars, where available, represent the standard deviation in measurements.

Ti-6Al-4V undergoes a transformation from the body centered cubic β-phase to a two phase structure consisting primarily hexagonally close-packed α-phase and small amounts of β-phase at a temperature of approximately 1000 °C [16]. The solid-state transformation can lead to quantifiable microstructural features within grains and depending on the cooling rate through transition temperature, the α-phase can exhibit different morphologies. In many AM builds, needle-like α-laths are present inside the large, prior β grains. Multiple studies have investigated the quantitative relationship between α-lath width and mechanical properties like hardness, strength and ductility. A collection of measured data correlating α-lath width to Vickers hardness is presented in Figure 19, which shows a decrease in hardness with the coarsening of the lath for DED-L and PBF-EB.

Heat treatments are used to relieve residual stresses and coarsen α-phase morphology for increasing ductility and toughness at the expense of strength and hardness in Ti-6Al-4V components fabricated by AM. A similar behavior was observed during the wire fed DED-L of Ti-6Al-4V that was stress relieved at 600 °C for 4 h. The Vickers hardness of multiple samples deposited with different process parameters (~327 HV) was found to increase to approximately 343 HV after stress relieving. It was suggested that a combination of precipitation hardening and solid solution strengthening contributed the increase, as the selected heat treatment temperature can also be used for aging and energy dispersive spectroscopy showed slightly less segregation compared to the as-deposited condition.

### 3.5. Grain Size

Grain size refining is known to have a significant impact on the strength and hardness of metals and alloys. The early works of Hall [180] and Petch [181,182] described the effect of grain size, *d*, on yield strength, *σ_y_*, in the well-known Hall-Petch relation,

(2) $$\sigma_{y}=\sigma_{0}+\frac{k_{y}}{\sqrt{d}}$$

where *σ*_0_ and *k_y_* are material constants that represent the yield stress of a grain-free material and the strengthening coefficient, respectively. The expression was formulated to explain the observed phenomenon that fine-grained materials exhibit higher stresses prior to yielding compared to alloys with coarse grains. At grain boundaries where there is a change in crystallographic orientation, dislocations require more energy to move from one grain to another, thereby impeding dislocation motion. Therefore, higher grain boundary area per unit volume (smaller grain size) effectively strengthens a material by blocking dislocation motion.

**Figure 20:**
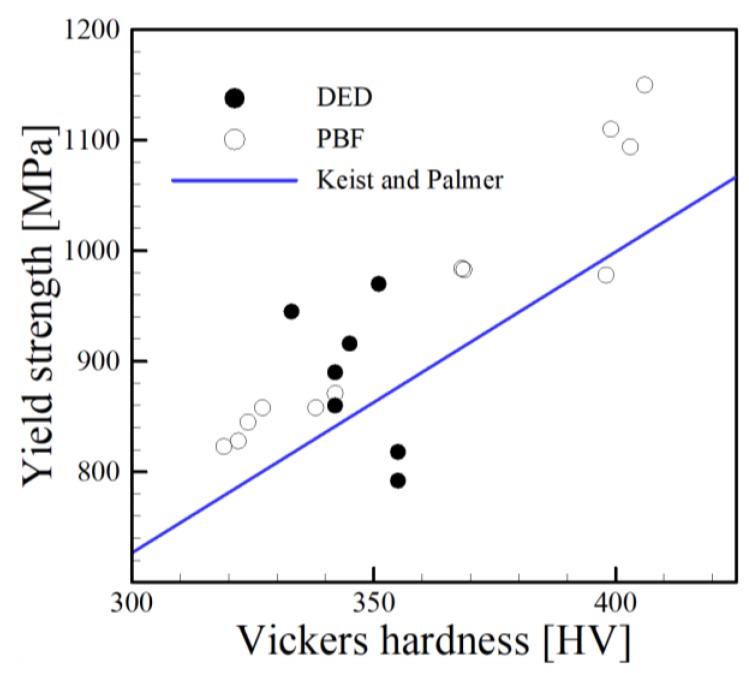
Comparison between the correlation developed by Keist and Palmer [1] and independent experimental data [54,55,57,58,59,185,186,187] for yield strength and hardness measurements spanning multiple AM processes.

**Figure 21:**
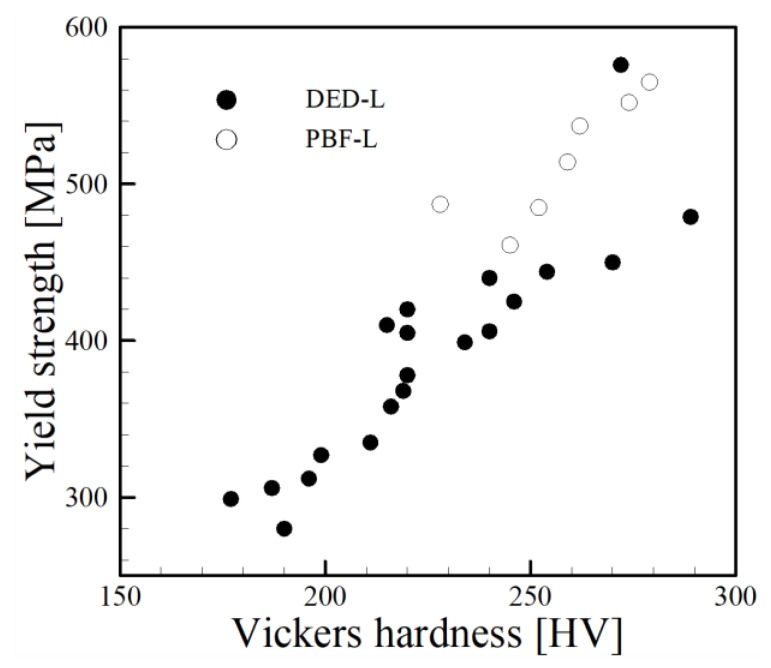
A collection of independent experimental data showing a comparison between measured yield strength and Vickers hardness for SS316L fabricated by AM [46,48,49,188,189].

The works of Tabor [183] and Cahoon [184] have shown that hardness is directly proportional to yield strength. Recently, Keist and Palmer [1] investigated the strength-hardness relationships for the DED of Ti-6Al-4V using both laser and electron beam heat sources. Their correlation is plotted along with independent experimental data for Ti-6Al-4V components fabricated by PBF and DED processes in Figure 20. Similarly, a collection of data for the AM of SS316L is shown in Figure 21. While scatter in data from multiple researchers can be expected, both Figure 20 and Figure 21 clearly show positive correlations between yield strength and hardness for AM alloys fabricated by multiple techniques. Therefore, Equation (2) can also be applied to studies involving the relationship between hardness and grain size. When considering the micro-hardness measurement using a Vickers indenter, the relationship takes a similar form, where yield strength is replaced by Vickers hardness, *HV* and *HV*_0_ is material constant reference hardness value replacing *σ*_0_. As hardness is a measurement of *localized* plastic deformation, a hardness indent may be fully encompassed within a grain depending on the load, dwell time and size of grains and the effect of strengthening due to dislocation propagation and pileup at grain boundaries may not be captured. Also, significant variability in measurements could result depending on where measurements are taken with respect to grains (center of grain, boundary, triple junction, etc.). Regardless, the acquisition of ample hardness measurements for many AM microstructures provides a simple means for investigating the effect of grain size on hardness.

The Hall-Petch relation was originally developed for equiaxed grains and has found good agreement with experimental results in the grain size range on the order of approximately a few to hundreds of microns. Grain sizes in AM alloys typically fall within this range, however many grains have a columnar rather than equiaxed morphology. Also, grain sizes in PBF processes exhibit smaller grain sizes compared to DED processes. In the AM of IN625 for example, Li et al. [190] measured grain sizes <40 μm after PBF-L while Khayat and Palmer [155] measured sizes in the hundreds of microns for DED-L. Typically, aspect ratios in AM, which are defined as the ratio of grain length to grain width, range between 1 (equiaxed) and 10 (elongated columnar). As-deposited microstructures usually exhibit lower aspect ratios than heat treated samples where significant growth along the length dimension can occur. As relationships between grain size and yield strength are commonly reported in AM literature, it is important for researchers to be specific about the grain dimension used for analysis.

**Figure 22:**
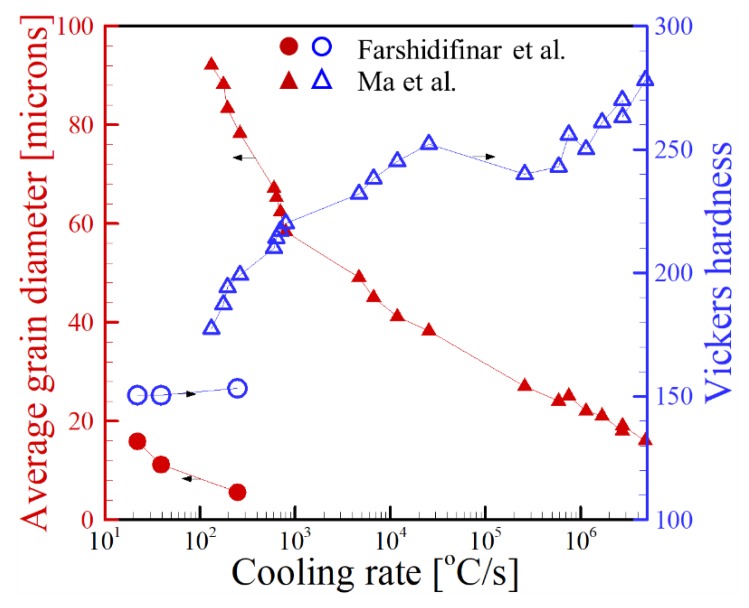
The relationship between cooling rate, average grain diameter and Vickers hardness for the AM of SS316L [88,188]. The cooling rate on the horizontal axis is plotted with a logarithmic scale and black arrows indicate the y-axis for each data set.

Since grain coarsening is a thermally activated process, the amount of time that an alloy remains at high temperatures ultimately determines the size of grains. Therefore, high cooling rates such as those encountered in PBF processes tend to yield small grain sizes while low cooling rates in high power DED processes exhibit larger grains. Experimental data [88,188] relating cooling rate to grain size and Vickers hardness for the AM of SS316L is shown in Figure 22. The data includes measurements from both DED-L and PBF-L processes at various combinations of laser power and scanning speed. In each study, the average grain diameter decreased with an increase in cooling rate. Consequently, an inverse relationship was obtained between Vickers hardness and average grain diameter, resulting in the Hall-Petch effect.

**Figure 23:**
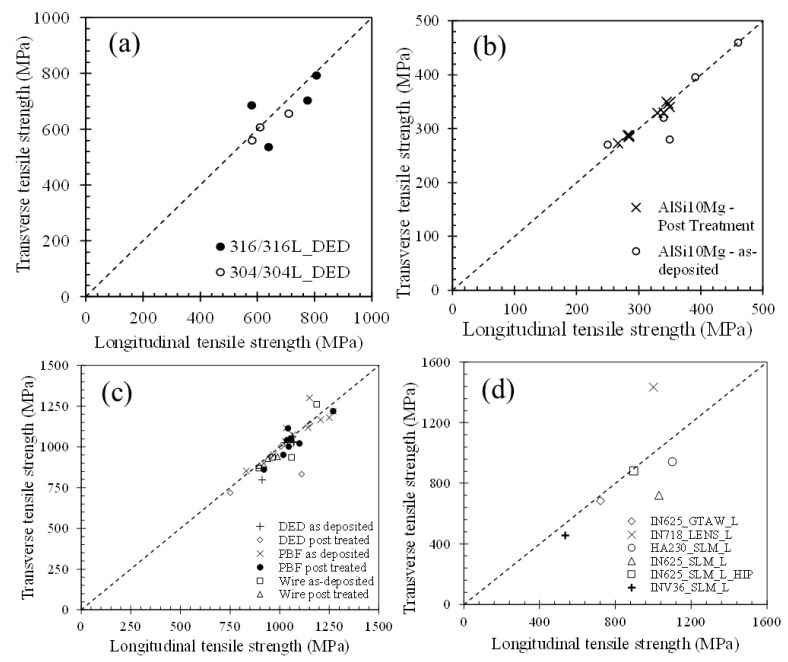
An analysis of anisotropic behavior [17] through a comparison between the transverse and longitudinal tensile strengths in additively manufactured (**a**) stainless steels [4,45,46,48,191], (**b**) aluminum alloy AlSi10Mg [105,106,192,193,194,195,196], (**c**) Ti-6Al-4V [54,57,89,197,198,199,200,201,202,203,204,205,206,207,208,209] and (**d**) nickel alloys [210,211,212,213,214,215]. Data points deviating from the dashed one-to-one line are exhibit more anisotropy compared to those lying close to the line. Reprinted from [17] with permission from Elsevier.

Wang et al. [4] studied the effects of processing conditions and microstructural features on the tensile properties of SS304L during DED-L. In their discussion, the grain dimension was defined as the average measured length of the grain in the direction of loading. It was found that the measured yield strength and grain sizes obeyed the Hall-Petch relation. Interestingly, the yield and tensile strengths measured in the transverse (parallel to the long grain axis) and longitudinal (parallel to the short grain axis) showed no clear anisotropic trends. In fact, a collection of literature data in a recent review [17] showed that although the microstructures of AM alloy components exhibit elongated columnar grains, the amount of anisotropy is negligible when comparing tensile properties in orthogonal directions. This finding is summarized in Figure 23 for different alloys and AM processes. Data points near the dotted lines in Figure 23 represent little anisotropy while those that stray from the line exhibit more anisotropic behavior.

### 3.6. Dislocations in AM Materials

**Figure 24:**
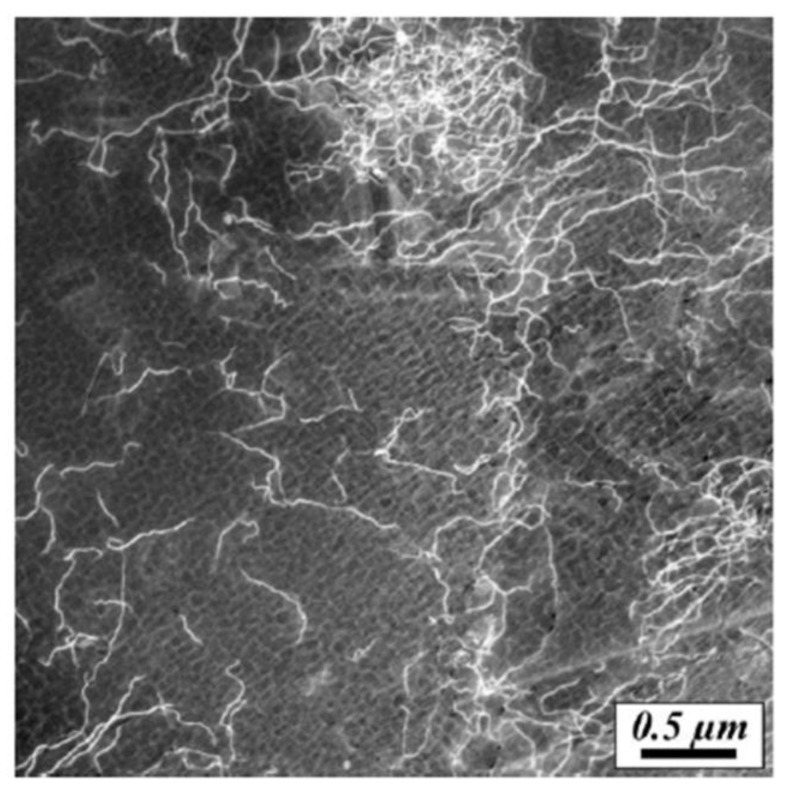
TEM micrograph of dislocations in nickel-based superalloy CMSX-4 processed by PBF-EB AM [217].

Although no investigations have directly examined the impact of dislocation structures on hardness in AM materials, it is important to discuss the role of dislocations on strengthening in general. The scale of dislocations requires experimental observation to be performed by transmission electron microscopy and Figure 24 shows an example of dislocations in a single crystal alloy fabricated by PBF-EB. In conventional metals processing, parts are often work hardened to achieve desired properties, which effectively uses plastic deformation to introduce a high density of dislocations. Dislocations can exist in many different forms such as edge, screw or mixed, where each introduces strain in the crystal lattice which affects the movement of neighboring dislocations during plastic deformation. In most introductory materials science and engineering textbooks, the tradeoff between strength and ductility is formulated in which any increase in strength due to work hardening is accompanied by a loss in ductility. In a recent work by Wang and co-workers [216], a hierarchy of microstructures spanning multiple orders of magnitude was attributed to help overcome the conventional strength-ductility tradeoff for PBF-L of SS316L. Very fine cellular walls with high dislocation densities and elemental segregation were found to pin dislocation motion and promote twinning, which ultimately lead to an increase in both strength and ductility due to a steady work hardening behavior.

**Figure 25:**
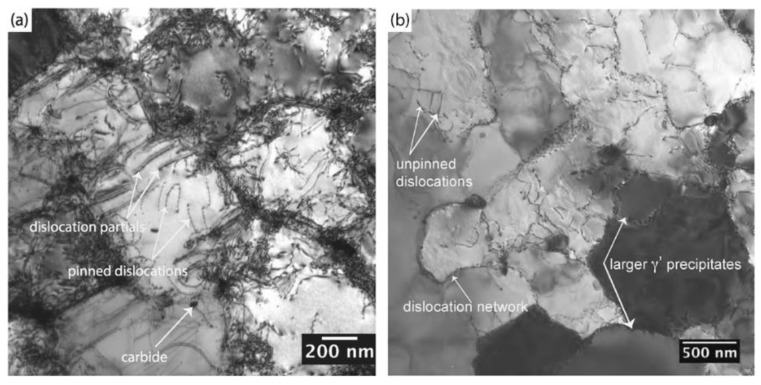
Dislocation structures in nickel-based superalloy CM247LC fabricated by PBF-L [218] showing (**a**) high dislocation density in the as-deposited condition, especially near cell edges and (**b**) reduced dislocation density after heat treatment at 1230 °C for 2 h followed by air cooling. Reprinted from [218] with permission from Elsevier.

In a recent review, Gorsse et al. [19] offered a calculation procedure to determine the upper limit of dislocation density (5 × 10^15^ m^−2^) for steels by assuming that all linear thermal strain is accompanied by dislocations upon cooling after solidification. This rough estimation is in good agreement with reported dislocation densities of AM materials in Table 3. Overall, the dislocation densities are comparable to those determined in wrought materials, which is uncharacteristic for materials that have not been work hardened. Moreover, the dislocations in AM materials are often organized into networks [218], as shown in Figure 25a. As in conventional materials, heat treatment will effectively lead to a reduction in dislocation density as shown in Figure 25b. Future research can take advantage of the unique dislocation structures in AM materials if the macroscopic process can be used to control the sub-micron microstructural features [216]

## 4. Compositional Variables

### 4.1. Iron Alloys

A particularly useful approach for predicting properties of steels that has been previously used in the welding community is through the carbon equivalent [226]. The carbon equivalent can take on many forms that include various alloying elements to best suit the target application. The HV for various iron-based alloys can be related to composition using the critical weldability (P_cm_), which was originally derived for evaluating crack susceptibility for a wide variety of alloy steels, given as [227]:

(3) $$P_{\mathrm{cm}}\;=\;C\;+\;\frac{\mathrm{Si}}{30}\;+\;\frac{\mathrm{Mn}+\mathrm{Cu}+\mathrm{Cr}}{20}\;+\;\frac{\mathrm{Ni}}{60}\;+\;\frac{\mathrm{Mo}}{15}\;+\;\frac{V}{10}\;+\;5B$$

All elements in Equation (3) are expressed in weight percent. The average HV values from independent experimental data [91,92,93,94,95,96,97,98,99,226] were plotted versus the P_cm_ of the alloy for the following ranges of alloying elements: 0.02–0.99 wt % C, 0–10.2 wt % Co, 0–13.3 wt % Cr, 0.2–1.62 wt % Mn, 0.06–7.97 wt % Mo, 0.15–18.8 wt % Ni, 0.011–0.025 wt % P, 0.29–1.02 wt % Si, 0–0.88 wt % Ti, 0.03–2.01 wt % V and 0–6.32 wt % W. Since different measurement methods such as the Rockwell C hardness test are used for steels, a conversion between the hardness scales is needed. The following relationship was used to convert from the Rockwell C (HRC) to the HV scale [228]:

(4) $${\mathrm{HV}\;=\;111e}^{0.0316(\mathrm{HRC})}$$

**Figure 26:**
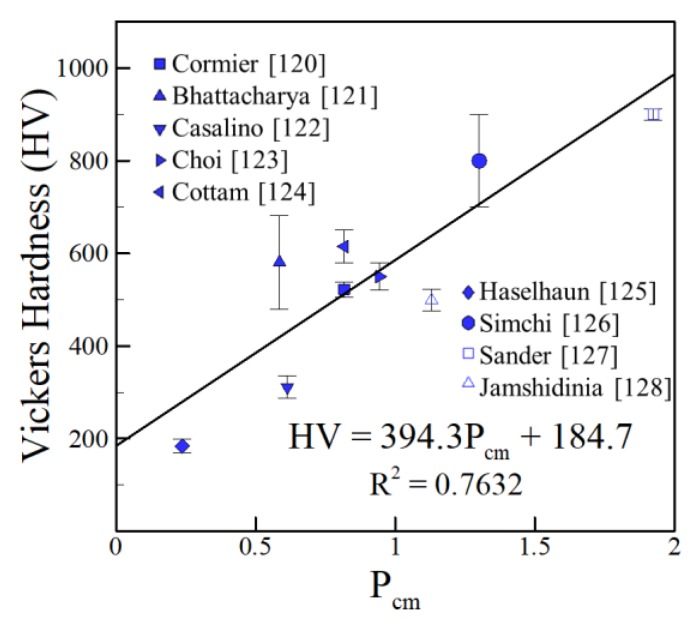
Experimentally measured hardness [91,92,93,94,95,96,97,98,99] vs. P_cm_ of iron-based alloys for AM.

Figure 26 shows that a linear fit is achieved between HV and composition for data from AM shown in Table 4. It is well-accepted that process variables affect hardness of steels in AM based on many factors that include microstructural features. However, the linearity of Figure 26 shows that chemical composition of steels can provide an approximate value of hardness independent of the AM process variables selected.

### 4.2. Aluminum Alloys

Aluminum alloys offer great opportunity for producing lightweight parts for aerospace and automotive applications. As pure aluminum is a relatively soft metal, these alloys often rely on alloying elements to achieve higher strength and hardness through solid solution strengthening and work hardening, such as in 5xxx series alloys, or precipitation hardening, as in the 6xxx and 7xxx series.

A constrained multi-variate linear regression analysis is used for determining the dependence of as-deposited hardness on composition for the AM of aluminum alloys. The following relationship between experimentally measured HV values and chemical composition was obtained where each element is in weight percent:

(5) HV = 37.99 + 19.47Ag + 2.85Cu + 23.36Fe + 24.47Mg+ 30.00Mn + 5.43Si + 20.86Ti + 19.06Zn

**Figure 27:**
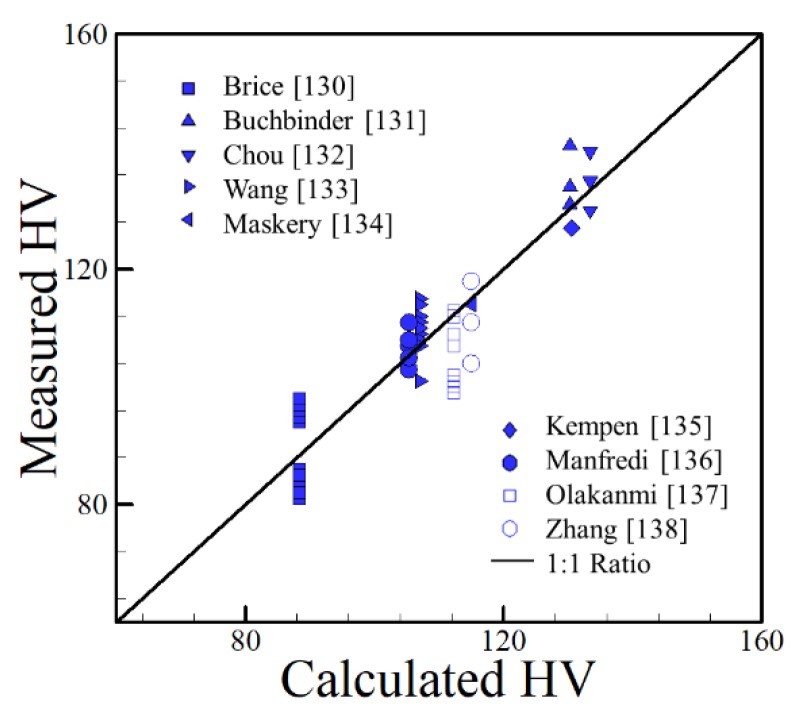
Experimentally measured [100,101,102,103,104,105,106,107,108] and calculated HV of aluminum alloys fabricated by AM using Equation (5).

The correlation presented is valid for the following ranges of alloying elements: 0–0.5 wt % Ag, 0–5.3 wt % Cu, 0–0.8 wt % Fe, 0–1.95 wt % Mg, 0–0.55 wt % Mn, 0–12.2 wt % Si, 0–0.064 wt % Ti and 0–0.1 wt % Zn. The data points with alloying elements that were well outside of the valid ranges mentioned above were omitted. The relationship between the measured hardness and the hardness calculated using Equation (5) for AM data [100,101,102,103,104,105,106,107,108] is shown in Figure 27. The solid line in the plot is the one-to-one relationship between measured and calculated values, meaning that a point falling on this line is exactly predicted by Equation (5). The chemical compositions are shown in Table 5.

### 4.3. Nickel Alloys

Nickel alloys are sought after for their excellent high temperature properties and corrosion resistance. Often, alloying elements can account for nearly 50% of the total weight of the alloy. Many nickel alloys are age-hardenable and under the appropriate heat treatment, they can exhibit numerous equilibrium phases consisting of solid solutions, intermetallic compounds and fine precipitates. However, it is often found that due to the high cooling rates encountered during AM, insufficient time is given for these secondary phases to nucleate and grow and many major alloying elements can remain in solid solution [15].

**Figure 28:**
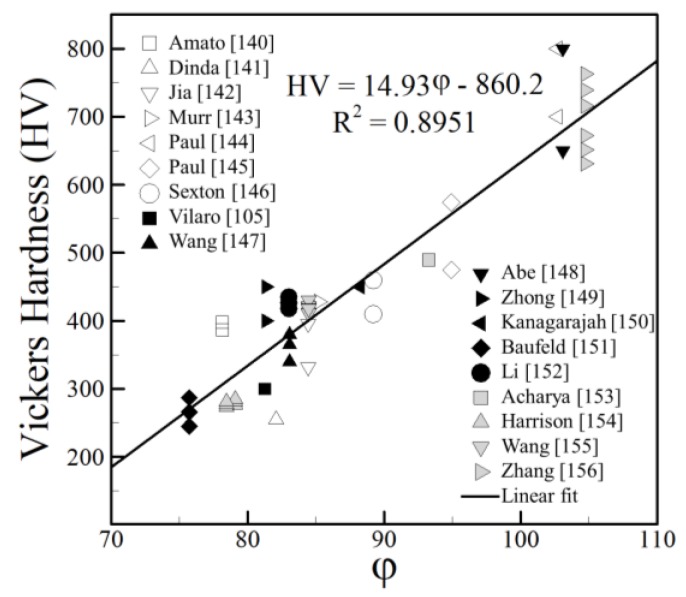
As-deposited HV [67,109,110,111,112,113,114,115,116,117,118,119,120,121,122,123,124,125] as a function of φ, which depends on the chemical composition of nickel-based AM alloys. Reprinted from [17] with permission from Elsevier.

A form of the nickel equivalent [229] is adopted, which is used as a guide for predicting austenite stability during high cooling rate processes, such as welding. The expression used is given as:

(6) $${\mathrm{Ni}}_{\mathrm{EQ}}\;=\;\mathrm{Ni}\;+\;0.65\mathrm{Cr}\;+\;0.98\mathrm{Mo}\;+\;1.05\mathrm{Mn}\;+\;0.35\mathrm{Si}\;+\;12.6C$$

where all elements are given in weight percentage. To include other alloying elements, a linear regression analysis can be applied to the remainder of elements that are not included in the Ni_EQ_ expression. The final term, which will be denoted as φ, can be expressed as:

(7) $${\varphi \;=\;\mathrm{Ni}}_{\mathrm{EQ}}\;-\;6.36\mathrm{Al}\;+\;3.80B\;+\;0.01\mathrm{Co}\;+\;0.26\mathrm{Fe}\;+\;7.06\mathrm{Hf}\;+\;1.20\mathrm{Nb}\;+\;4.95\mathrm{Ta}\;+\;5.78\mathrm{Ti}\;+2.88W$$

where all elements are given in weight percentage. The expression is valid in the following range of elements: 0–6.5 wt % Al, 0–3.75 wt % B, 0–0.5 wt % C, 0–19.2 wt % Co, 0–21.8 wt % Cr, 0–24.7 wt % Fe, 0–1.5 wt % Hf, 0–0.48 wt % Mn, 0–9.75 wt % Mo, 0–5.1 wt % Nb, 0–4.25 wt % Si, 0–6.35 wt % Ta, 0–4.7 wt % Ti and 0–4.9 wt % W. The compositions and HV values used are shown in Table 6. Only elements which either were common to the majority of the alloys considered or contributed to a significant portion of the alloy composition were considered in the analysis as they had negligible effects on the outcome. When independent HV measurements [67,109,110,111,112,113,114,115,116,117,118,119,120,121,122,123,124,125] are plotted against *φ* for various nickel alloys, the trend is linear as shown in Figure 28.

### 4.4. Comparison with Other Processes

**Figure 29:**
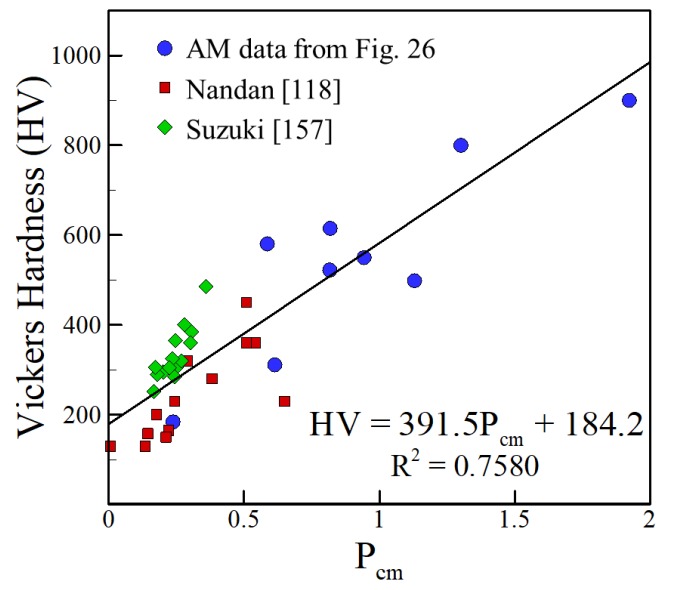
Comparison between measured as-deposited hardness values of iron-based alloys in AM, TMAZ hardness values of FSW and as-welded hardness from fusion welding.

It is often useful to compare microstructures and properties with the cooling rates of various processes. Since AM is a rapidly emerging field and the underlying science is still being understood, it can be beneficial to compare the hardness of AM alloys with measurements from other processes. Let us consider fusion welding, where melting is used for joining and friction stir welding (FSW), a process where solidification is not involved and joining is performed in solid state. To understand the relative role of manufacturing process variables and the chemical composition of steels, Figure 29 combines hardness data for steels from AM, FSW and fusion welding literature. Table 4 shows the specific compositions for AM while the compositions and hardness values for FSW and fusion welding can be found elsewhere [226,230,231]. To give the reader a sense of the cooling rates, Table 7 shows a collection of available data for cooling rates measured in the three processes. There are many orders of magnitude difference between the processes, ranging from reported values of 3 K/s for friction stir welding to 10^4^ K/s for AM.

Similar analyses for the hardness of steels in the thermo-mechanically affected zone (TMAZ) of FSW and the heat affected zone (HAZ) of fusion welding were presented by Nandan et al. [226] and Suzuki [230]. When comparing their work to Figure 29, striking similarities are observed. Combining the data from AM, FSW and fusion welding, the HV of steels is linearly dependent on P_cm_ with R^2^ = 0.7580, which is slightly lower than the value of R^2^ = 0.7632 obtained for only AM data alone indicating consistency in the relationships. Figure 29 shows that when hardness is calculated for the TMAZ of FSW and the HAZ of fusion welding using Equation (3), a good correlation with a trend consistent to the AM data is obtained. It is important to note that the data from Ito and Bessyo [231] for which P_cm_ was originally derived for was clustered around small P_cm_ values of less than 0.4 that resulted in a large slope of the P_cm_ versus HV plot as shown by Suzuki [230]. As this work extends to a much broader range of P_cm_ values up to almost 2, a smaller slope is obtained. These findings show that the presented approximations can be applied to three different types of joining processes while still producing consistent results, highlighting the important role of composition for predicting hardness of steels.

**Figure 30:**
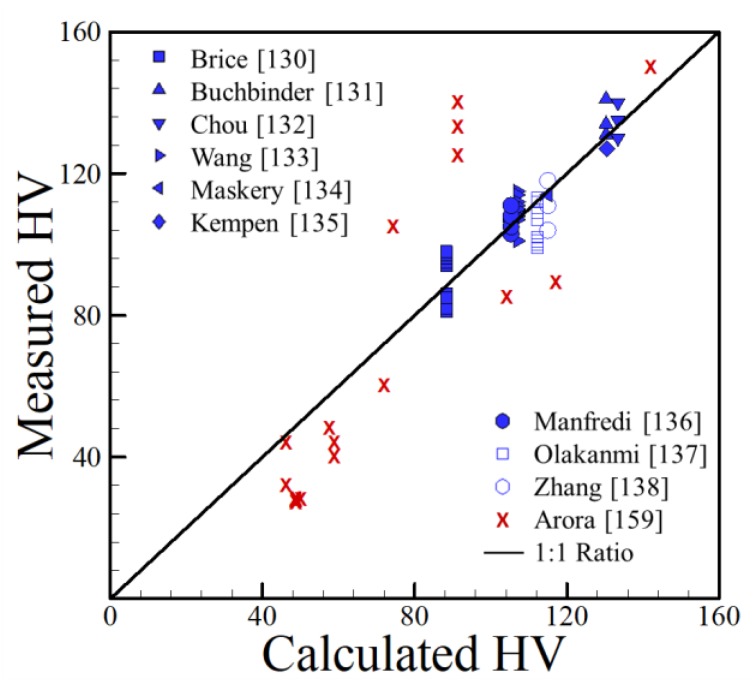
Comparison between measured and calculated hardness values using Equation (5) for AM and FSW data [242].

Data for the measured and predicted as-deposited hardness of FSW aluminum alloys is also compared to AM data in Figure 30. A similar plot for the hardness of aluminum alloys were presented by Arora et al. [242] for the FSW of aluminum alloys. Figure 30 shows that when as-welded hardness is calculated for the TMAZ of FSW using Equation (5), a good correlation with a trend consistent to the AM data is obtained. These findings show that Equation (5) can be applied to both AM and FSW while ignoring process variables and is still capable of producing approximate hardness values, signifying the importance of chemical composition in determining the hardness of aluminum alloys. For the FSW data, Mg and Zn were important alloying elements for the alloys considered, which is not the case here.

## 5. Concluding Remarks

The role of cooling rate, microstructure and alloy composition on the published hardness data of iron, aluminum, titanium and nickel alloy components fabricated by AM are examined. The correlations presented in this review provide a useful and practical means of obtaining an approximate value of hardness of AM alloys by conducting back-of-the envelope calculations. When the available data are critically reviewed, the following important conclusions that cannot be made from individual papers become apparent.

(a) The variations of process parameters and cooling rates change microstructures of AM alloys. However, when the influence of AM process parameters on hardness is evaluated from the reported independent hardness values of fabricated components prior to post-deposition heat treatment, the variation of hardness falls within a narrow band of values for ferrous, aluminum and nickel alloys.
(b) The range of hardness variation of AM alloys in the as-fabricated state attainable by varying AM process parameters is much lower than the hardness enhancement attainable by subsequent heat treatment or aging. The extent of hardness variation by varying AM process variables is roughly the same as the reported variations of hardness of iron and aluminum alloy weld metals when welding parameters are varied.
(c) The reported hardness data show approximate linear trends with appropriate compositional variables for iron, aluminum and nickel alloys over a wide range of AM variables and processes. The scatter in the hardness data for all alloy systems fall within a small band of values that correlates well with the concentration of alloying elements. The correlations developed are approximate and valid for the range of composition indicated but the findings are consistent over a wide range of processes and process parameters.
(d) Although AM offers many advantages in fabricating metallic components, a target hardness of components in as fabricated condition is much more easily obtained by alloy selection rather than by changing AM processing variables. In this sense, the AM produced materials behave in a manner similar to other conventional metals processing technologies.

## Figures and Tables

**Table 1:** Process parameters for iron, aluminum and nickel alloys.

Alloy		* Process	^†^ Power (W)	Scanning Speed (mm/s)	Ref.
Iron Alloys	H13	EBM	(20 mA)	500	[91]
	4340	DMD	500	7.5	[92]
	18Ni300	SLM	86–100	180–220	[93]
	H13	DMD	1000–1400	10.5–19.0	[94]
	H13	DMD	2500	5	[95]
	ER70S-6	3D GMAW	(14–16 V, 65–76A)	5.23	[96]
	M2 steel	DMLS	200	50–175	[97]
	Tool steel	SLM	75–175	300-450	[98]
	420 SS	DMLS	283–317	600–1000	[99]
Aluminum Alloys	Al 2139	EBF^3^	1350	8.47	[100]
	AlSi10Mg	SLM	195	800	[101]
	Al-12Si	Pulsed SLM	500–4500	1.5–3.0	[102]
	Al-12Si	SLM	200	37–2000	[103]
	AlSi10Mg	SLM	200	318	[104]
	AlSi10Mg	SLM	200	Not reported	[105]
	AlSi10Mg	DMLS	120	900	[106]
	Al-12Si	SLS	100–200	80–200	[107]
	Al 2024	SLM	200	83–333	[108]
Nickel Alloys	IN718	SLM	200	800–1200	[109]
	IN718	DMD	750	6.25	[110]
	IN718	SLM	110–130	400–600	[67]
	Rene 142	EBM	Not reported	Not reported	[111]
	Colmonoy 6	LRM	2500	4.2	[112]
	IN625	LRM	1000–1500	5.0–13	[113]
	Rene 142	LC	550	Not reported	[114]
	Nimonic 263	SLM	200	100	[115]
	IN718	SLM	170	417	[116]
	Experimental	SLM	1000	2	[117]
	IN718	SLM	3000	Not reported	[118]
	IN939	SLM	400	540–620	[119]
	IN718	SMD	(220A)	5	[120]
	Rene 41	LMD	4500–5000	5.00–5.83	[121]
	Rene 80	SLE	1000	~100	[122]
	Hastelloy X	SLM	165–195	Not reported	[123]
	Hastelloy X	SLM	Not reported	Not reported	[123]
	IN718	SLM	Not reported	Not reported	[124]
	Ni60A	LMDS	Not reported	Not reported	[125]

* EBM = Electron beam melting, DMD = Direct metal deposition, SLM = Selective laser melting, 3D-GMAW = Gas metal arc welding 3D printing, DMLS = Direct metal laser sintering, EBF^3^ = Electron beam freeform fabrication, DMLS = Direct metal laser sintering, SLS = Selective laser sintering, LRM = Laser Rapid Manufacturing, LC = Laser Cladding, SMD = Shaped Metal Deposition, LMD = Laser Metal Deposition. ^†^ Values in parenthesis signify processes where it is more common to report the voltage and current.

**Table 2:** Process and resulting as-deposited microstructures for iron, aluminum and nickel alloys.

Alloy		Process	Phases	HV	Ref.
Iron alloys	H13	EBM	Martensite	^‡^ 498.5 ± 14.5	[91]
	4340	DMD	Ferrite, Martensite, Cementite	580.5 ± 100.5	[92]
	18Ni300 Maraging steel	SLM	Not reported	^‡^ 323.5 ± 21.5	[93]
	H13	DMD	Not reported	550 ± 30	[94]
	H13	DMD	Fine martensite, retained austenite, fine carbides	615 ± 35	[95]
	ER70S-6	3D-GMAW	Polygonal ferrite, acicular ferrite	184 ± 15	[96]
	M2 steel	DMLS	Martensite, austenite, fine carbides	800 ± 100	[97]
	FeCrMoVCtool steel	SLM	Fine martensite, austenite, carbides	900 ± 12	[98]
	420 SS	DMLS	Martensite, little retained austenite	^‡^ 478 ± 20	[99]
Aluminum alloys	Al 2139	EBF^3^	Not reported	81–103	[100]
	AlSi10Mg	SLM	FCC dendrites, interdendritic eutectic	131–141	[101]
	Al-12Si	Pulsed SLM	FCC dendrites, small eutectic phases	130–140	[102]
	Al-12Si	SLM	FCC Al matrix, nanosized Si precipitates	107–115	[103]
	AlSi10Mg	SLM	FCC	114	[104]
	AlSi10Mg	SLM	FCC, fine Si precipitates	127	[105]
	AlSi10Mg	DMLS	FCC	103–111	[106]
	Al-12Si	SLS	FCC Al-Si matrix	99–113	[107]
	Al 2024	SLM	FCC	104–118	[108]
Nickel alloys	IN718	SLM	FCC-γ, ellipsoidal Ni3Nb precipitates	387–398	[109]
	IN718	DMD	FCC-γ	255	[110]
	IN718	SLM	FCC-γ, fine γ’ precipitates	331.9–395.8	[67]
	Rene 142	EBM	FCC-γ, cuboidal γ’ precipitates	428.1	[111]
	Colmonoy 6	LRM	FCC-γ, interdendritic eutectic	700–800	[112]
	IN625	LRM	FCC-γ	^‡^ 474.9–574.1	[113]
	Rene 142	LC	Not reported	410–460	[114]
	Nimonic 263	SLM	γ, carbides in interdendritic regions	300	[115]
	IN718	SLM	FCC-γ	340–380	[116]
	Experimental	SLM	Not reported	650–800	[117]
	IN718	SLM	FCC-γ	400–450	[118]
	IN939	SLM	FCC-γ	450	[119]
	IN718	SMD	FCC-γ, interdendritic carbides/Laves	245–287	[120]
	Rene 41	LMD	FCC-γ, MC carbides	418.1–435.1	[121]
	Rene 80	SLE	FCC-γ, fine carbides/γ’ particles	489.8	[122]
	Hastelloy X	SLM	FCC-γ	276.9–284.9	[123]
	Hastelloy X	SLM	FCC-γ	273.2–281.0	[123]
	IN718	SLM	FCC-γ	410.8–430.2	[124]
	Ni60A	LMDS	FCC-γ	^‡^ 631.1–762.9	[125]

^‡^: Converted from HRC to HV using Equation (4).

**Table 3:** A summary of dislocation densities reported for AM alloys.

Alloy	Process	Dislocation Density (m^−2^)	Reference
SS316L	PBF-L	1.5 × 10^14^	[219]
SS316L	PBF-L + Solutionized	9.7 × 1013	[219]
SS316L	Hot worked + Solutionized	3.5 × 10^13^	[219]
SS316L	DED-L	2.77 × 10^14^	[220]
SS304L	DED-L	4.31 to 7.45 × 10^12^	[220]
SS304L	PBF-EB	2.72 × 10^14^	[220]
SS304L	Wrought	1.84 × 10^14^	[220]
CrMnFeCoNi	DED-L	0.89 to 1.19 × 10^14^	[221]
IN718	PBF-L	2.00 × 10^13^ to 5.62 × 10^15^	[222]
Nb	PBF-EB	10^13^ to 10^14^	[223]
Ti-6Al-4V	DED-EB + HIP	10^11^	[224]
Ti-6Al-4V	DED-EB + Stress relieved	10^15^	[224]
SS304L	PBF-L	(3.8 ± 1) × 10^14^	[225]
SS304L	DED-L	(2.5 ± 1) × 10^14^	[225]
SS304L	Wrought (deformed)	(6.8 ± 1) × 10^14^	[225]

**Table 4:** Compositions (in wt %), P_cm_ values and average HV numbers for iron-based alloys.

Alloy	C	Co	Cr	Cu	Mn	Mo	Ni	P	Si	Ti	V	W	P_cm_	Average HV	Ref.
H13	0.37	-	4.99	-	0.2	1.1	-	0.011	1.02	-	0.8	-	0.817	^‡^ 498.5 ± 14.5	[91]
4340	0.42	-	0.9	-	0.74	0.45	2.63	-	0.29	-	-	-	0.586	580.5 ± 100.5	[92]
18Ni300 Maraging steel	0.02	10.2	-	-	-	4.2	18.8	-	-	0.88	-	-	0.613	^‡^ 323.5 ± 21.5	[93]
H13	0.47	-	5.01	-	0.2	1.2	-	-	0.63	-	1.12	-	0.944	550 ± 30	[94]
H13	0.35	-	5	-	0.35	1.5	-	-	-	-	1	-	0.818	615 ± 35	[95]
ER70S-6	0.1	-	0.15	-	1.62	0.15	0.15	0.025	1	-	0.03	-	0.237	184 ± 15	[96]
M2 steel	0.86	-	1.25	-	0.37	5.23	-	-	0.33	-	-	6.32	1.301	800 ± 100	[97]
FeCrMoVCtool steel	0.99	-	4.02	-	-	7.97	-	-	-	-	2.01	-	1.923	900 ± 12	[98]
420 SS	0.42	-	13.3	-	0.33	0.06	0.37	-	0.54	-	-	-	1.130	^‡^ 478 ± 20	[99]

^‡^ Converted from HRC to HV using Equation (4).

**Table 5:** Compositions (in wt %) and HV ranges used for aluminum-based alloys.

Alloy	Ag	Cu	Fe	Mg	Mn	Si	Ti	Zn	HV	Ref.
Al 2139	0.5	5.3	0.08	0.52	0.31	0.051	0.064	-	81–103	[100]
AlSi10Mg	-	-	0.55	0.4	0.45	10	-	0.1	131–141	[101]
Al-12Si	-	0.3	0.8	0.1	0.15	12	-	0.2	130–140	[102]
Al-12Si	-	0.003	0.12	-	-	12.2	-	-	107–115	[103]
AlSi10Mg	-	0.05	0.25	0.4	0.1	10	0.1	0.1	114	[104]
AlSi10Mg	-	0.1	0.55	0.4	0.45	10	-	0.1	127	[105]
AlSi10Mg	-	0.001	0.16	0.35	0.002	10.08	0.01	0.002	103–111	[106]
Al-12Si	-	0.08	0.36	-	-	12.1	-	-	99–113	[107]
Al 2024	-	4.47	-	1.95	0.55	-	-	-	104–118	[108]

**Table 6:** Compositions (in wt %) and range of HV numbers for nickel alloys.

Alloy	Al	Co	Cr	Fe	Mo	Nb	Si	Ti	C	Other	HV	Ref.
IN718	0.5	1.0	19.0	22.0	3.0	5.0	-	1.0	-	-	387–398	[109]
IN718	0.5	-	19.0	22.0	3.0	5.0	-	1.0	-	-	255	[110]
IN718	0.3	-	18.4	17.7	4.2	5.1	-	0.9	0.08	-	331.9–395.8	[67]
Rene 142	6.15	12.0	6.8	-	1.5	5.1	-	0.9	0.12	0.02B-1.5Hf-6.35Ta-4.9W	428.1	[111]
Colmonoy 6	-	0.24	13.6	4.75	-	-	4.25	-	0.6	2.5B	700–800	[112]
IN625	0.4	1.0	21.3	5.0	9.2	1.8	0.5	0.4	0.1	1.8Ta	^‡^ 474.9–574.1	[113]
Rene 142	3.0	9.5	14.0	0.1	3.8	0.03	0.01	5.0	0.14	0.02B-0.01Hf-0.01Mn-0.01Ta	410–460	[114]
Nimonic 263	0.5	19.2	19.5	0.5	6.0	-	0.2	2.4	-	-	300	[115]
IN718	0.29	-	18.2	18.9	3.1	5.1	-	0.9	0.03	-	340–380	[116]
Experimental	-	-	9.4	2.0	-	-	2.8	-	0.4	1.8B	650–800	[117]
IN718	0.41	-	15.9	17.1	1.9	2.23	-	1.27	-	0.31W	400–450	[118]
IN939	1.9	19.0	22.4	-	-	1.0	-	3.7	0.15	0.01B-1.4Ta-2.0W	450	[119]
IN718	-	-	19.0	24.7	3.0	-	0.35	-	0.08	0.35Mn	245–287	[120]
Rene 41	1.6	11.0	19.0	5.0	9.75	-	0.5	3.25	0.09	0.01B-0.5Mn	418.1–435.1	[121]
Rene 80	3.0	9.0	14.0	-	4.0	-	-	4.7	0.16	0.02B-0.8Hf	489.8	[122]
Hastelloy X	-	1.77	21.8	18.6	9.4	-	0.31	-	0.05	0.22Mn-1.05W	276.9–284.9	[123]
Hastelloy X	-	1.04	21.3	19.5	9.0	-	0.32	-	0.06	0.48Mn-0.56W	273.2–281.0	[123]
IN718	-	-	18.3	18.9	2.0	4.6	-	0.83	-	-	410.8–430.2	[124]
Ni60A	-	-	16.5	8.0	-	-	4.25	-	0.75	3.75B	^‡^ 631.1–762.9	[125]

^‡^ Converted from HRC to HV using Equation (4).

**Table 7:** Reported cooling rates for FSW, fusion welding and AM.

Process	Values/Ranges (K/s)	Reference
FSW	~5	[232]
FSW	~3 to 5	[233]
FSW	~90 to 120	[234]
FSW	~10	[235]
Submerged FSW	~20	[236]
Fusion welding	~5	[237]
Laser welding	10^0^ to 10^5^	[238]
Fusion welding	~10^3^	[227]
DED-L	4500	[239]
PBF-L	10^3^ to 10^4^	[197]
DED-L	10^4^	[240]
DED-L	~10^3^–10^4^	[9]
PBF-L	1.0 × 10^6^ to 4.0 × 10^7^	[241]
